# Recent Advances in the Enantioselective Organocatalytic [4+2] Cycloadditions

**DOI:** 10.3390/molecules30091978

**Published:** 2025-04-29

**Authors:** Tomasz Bauer

**Affiliations:** Faculty of Chemistry, University of Warsaw, L Pasteura 1, PL 02-093 Warsaw, Poland; tbauer@chem.uw.edu.pl

**Keywords:** [4+2] cycloaddition, Diels–Alder reaction, enantioselective synthesis, chiral phosphoric acid, proline-derived catalyst, Cinchona alkaloids, thiourea, squaramide

## Abstract

This review covers the recent advances in asymmetric organocatalytic Diels–Alder reactions published since the beginning of 2015. It describes recent approaches to enantioselective [4+2] cycloadditions based on the application of various types of chiral organocatalysts.

## 1. Introduction

An asymmetric Diels–Alder (DA) reaction is one of most valuable tools in asymmetric synthesis. Up to four stereogenic centers can be created in a single reaction step and the Diels–Alder adducts can be further elaborated to yield fundamental substrates for the synthesis of numerous natural products.

In the second half of the previous century, an approach based on the use of substrate- or chiral auxiliary-induced asymmetric synthesis and then chiral catalysis dominated reports [1]. In most cases, reactions were facilitated by the presence of Lewis acids as catalysts, achiral or chiral, which were often a significant problem, especially in pharmaceutical applications, because LAs are difficult to recover and are often toxic metals, dangerous for the experimenter and for the environment. Moreover, the majority of LA catalysts are highly water-sensitive; therefore, special precautions must be taken during experiments.

The development of organocatalysis was a game changer, and after the first publication on it [2], the enantioselective organocatalytic Diels–Alder method quickly became a worthy successor to the methods based on metal catalysis.

The aim of this review is to present to the reader the results achieved in the last 10 years, starting from 2015, while the results prior to this can be found in several excellent reviews on this topic [3,4].

## 2. Organocatalytic Diels–Alder Reactions

### 2.1. Secondary Amines as Catalysts

In this section, the results obtained using Hayashi’s [5] and Jørgensen’s [6] catalysts and their modifications and analogues will be discussed.

Earlier results have been reviewed by Jorgensen and co-workers [7]. The mechanism of action for both types of catalysts was thoroughly investigated by Hayashi [8].

**Figure 1:**
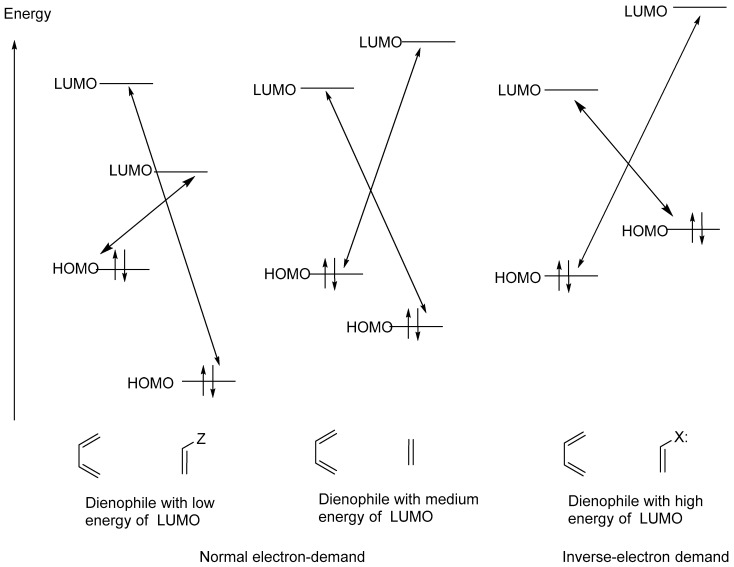
HOMO–LUMO interactions in Diels–Alder reaction.

Let us start with some general introduction to Diels–Alder reactions. The reactivity of both diene and dienophile in this process can be explained by the interactions of frontier orbitals. Classical [4+2] cycloaddition, in another words, a normal electron-demand Diels–Alder reaction, is governed by the interactions of the electron-rich diene’s HOMO with the electron-poor dienophile’s LUMO. The lower the energy of LUMO, the higher the reactivity of the dienophile and the faster the reaction. This is usually achieved by Lewis acid catalysis. In inverse electron-demand [4+2] cycloaddition, the electron-rich dienophile with higher HOMO energy interacts with the electron-poor diene’s LUMO, as presented in Figure 1.

**Figure 2:**
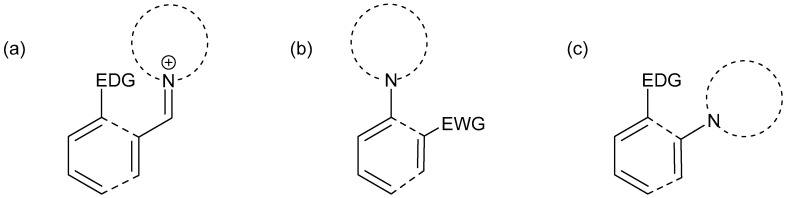
Three ways to activate reactants by amine: (**a**) iminium ion formation, (**b**) and (**c**) enamine formation.

The activation of the reactants in organocatalytic cycloadditions is achieved by the formation of iminium ions or enamines. The first approach is based on the pioneering work of MacMillan, in which the formation of the iminium ion intermediate, not the addition of a Lewis acid, lowers the LUMO’s energy and facilitates a normal electron-demand Diels–Alder reaction (Figure 2a). The presence of a strong acid, e.g., CF_3_COOH, as a co-catalyst accelerating iminium ion formation is usually required. The formation of enamine can be used in normal electron-demand Diels–Alder reactions, leading to the high-energy HOMO of dienamine or trienamine (Figure 2b), or in inverse electron-demand Diels–Alder reactions, raising the HOMO of the dienophile (Figure 2c).

**Scheme 1:**
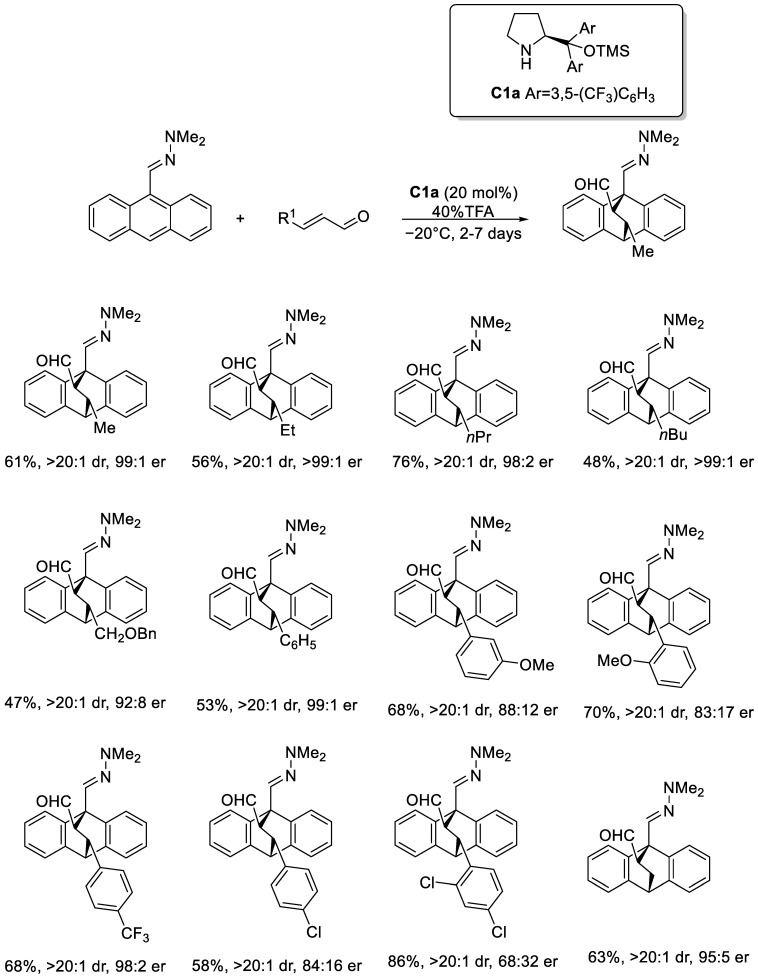
Addition of α,β-unsaturated aldehydes to hydrazone-substituted anthracenes.

Albrecht and co-workers described additions of α,β-unsaturated aldehydes to hydrazone-substituted anthracenes [9]. This system has the advantages of HOMO rising by the hydrazone moiety as an electron-donating substituent and LUMO lowering by iminium ion formation. The presence of water also had a beneficial effect on the reaction, once again confirming the advantage of organocatalysis over Lewis acid catalysis. Cycloaddition in the presence of Jorgensen’s catalyst **C1a** led to adducts with very high diastereo- and enantioselectivity (Scheme 1). The hydrazone moiety could be easily converted to a useful formyl or nitrile group. The chain length of the α,β-unsaturated aldehyde did not influence the selectivity of the rection; however, the substitution pattern of the phenyl ring highly influenced the observed enantiodiscrimination. The *ortho* substituents had a deteriorating effect on the selectivity.

**Figure 3:**
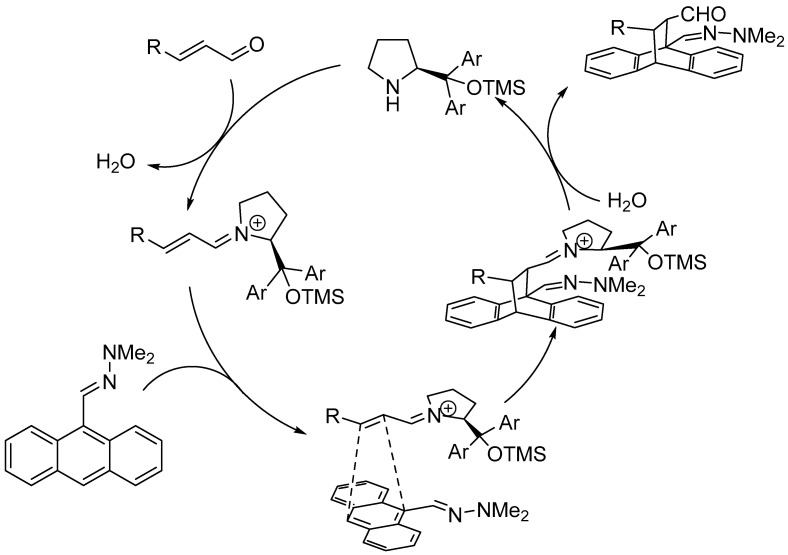
Possible mechanism of the reaction catalyzed by Jorgensen’s catalyst.

The mechanism of the reaction was proposed (Figure 3).

**Scheme 2:**
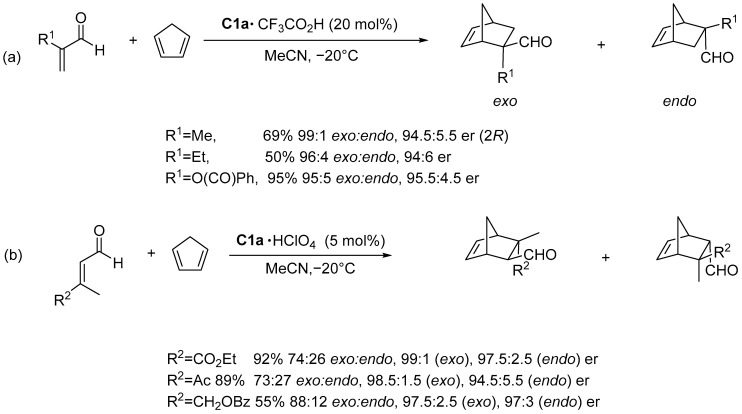
Diels–Alder reactions of α-substituted (**a**) and β,β-disubstituted α,β-unsaturated aldehydes (**b**).

Hayashi and co-workers investigated [4+2] cycloaddition to α-substituted (Scheme 2a) and β,β-disubstituted α,β-unsaturated aldehydes (Scheme 2b) [10]. The reaction in the presence of diarylprolinol **C1a** proceeded with a high *exo*:*endo* and enantioselectivity up to 97:3 er; however, no reaction was observed for α,β-disubstituted aldehydes.

The outcome of the reaction was highly dependent on the composition of the catalyst. The addition of α-substituted aldehydes went smoothly in the presence of the trifluoroacetic acid salt of **C1a**, but poor results were observed for β,β-disubstituted α,β-unsaturated aldehydes. Much better results were obtained for the perchloric acid salt of **C1a**.

**Scheme 3:**
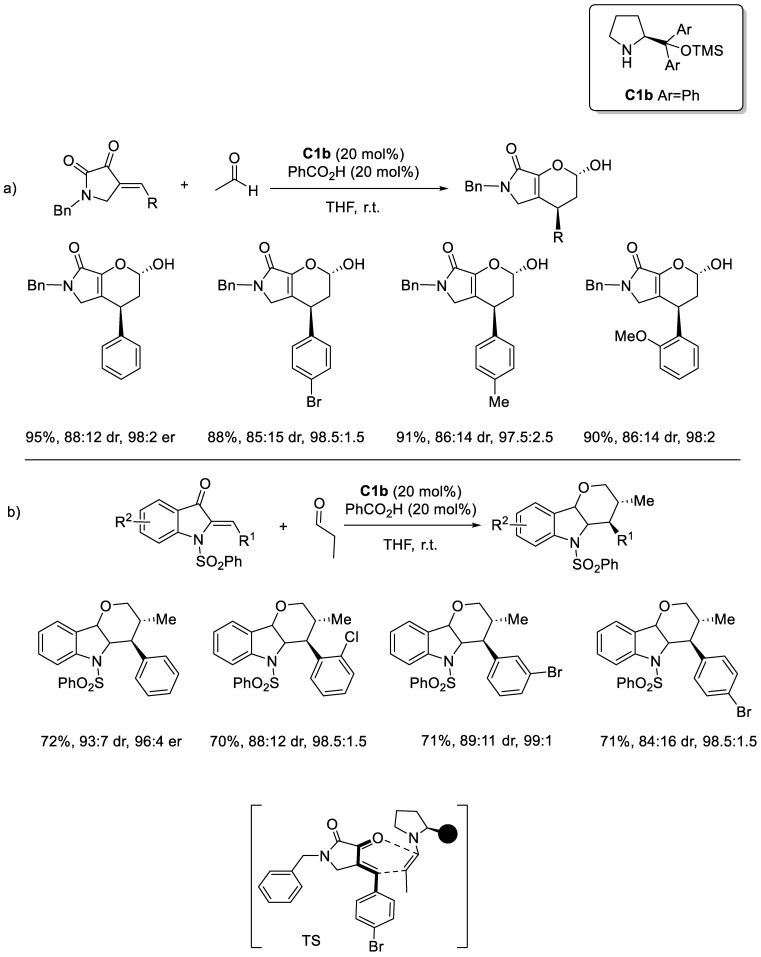
The inverse electron-demand Diels–Alder reactions of (**a**) acetaldehyde and (**b**) propanal.

Hayashi’s catalyst, **C1b**, was used for the first organocatalyzed reaction of aqueous acetaldehyde as the dienophile in an organocatalytic inverse electron-demand DA reaction with a heterocyclic enone-bearing exocyclic double bond (Scheme 3a). The same approach was applied for the reaction of propanal with (*Z*)-2-ylideneoxindoles (Scheme 3b). Both reactions proceeded with good chemical yields and excellent enantioselectivities. This oxo-inverse electron-demand Diels–Alder reaction takes advantage of enamine formation, leading to the increased energy of the dienophile’s HOMO (see TS in Scheme 3) [11,12]. The selected examples are presented in Scheme 3.

**Scheme 4:**
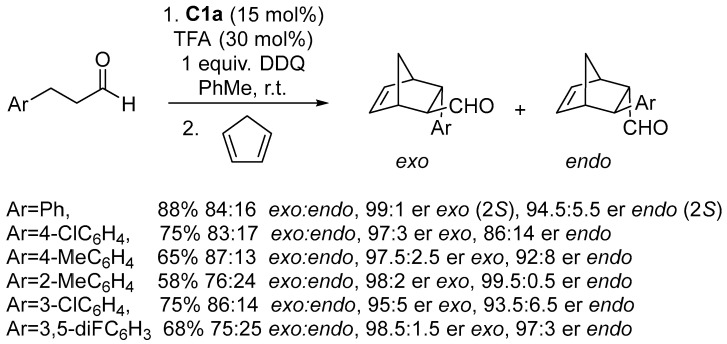
Enamine C-H oxidation/Diels–Alder reaction.

**Figure 4:**
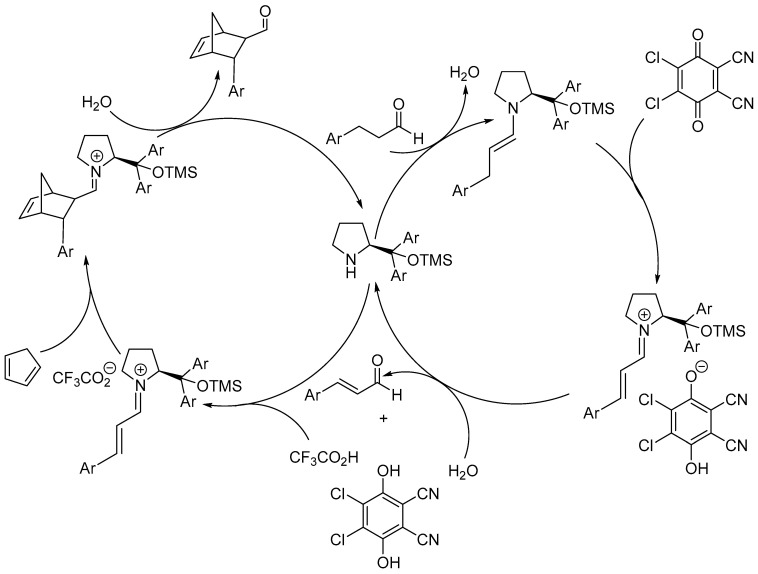
Mechanism of enamine C-H oxidation/Diels–Alder reaction.

Bondžić and co-workers proposed another approach, which allows for the use of saturated aldehydes as dienophiles in organocatalyzed DA reactions. The idea was based on the oxidation of an initially formed enamine to an iminium cation. Thus, the low-LUMO chiral dienophile formed was used in [4+2] cycloaddition to yield respective adducts with very good to excellent enantioselectivities [13]. Acyclic dienes as isoprene and 2,3-dimethylbuta-1,3-diene were unreactive under these conditions. 5-Aryl-substituted pent-4-enals could also be used as dienophiles with very good enantioselectivities up to 97:3 er. Selected results are presented in Scheme 4. The mechanism of this sequential reaction was proposed (Figure 4).

**Scheme 5:**
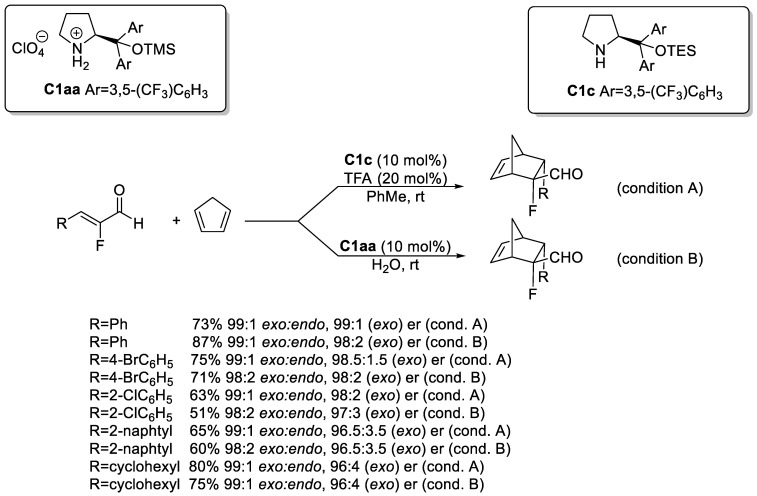
Addition of 2-fluoro-α,β-unsaturated aldehydes to cyclopentadiene.

The Diels–Alder reaction of 2-fluoro-α,β-unsaturated aldehydes with cyclopentadiene leading to the formation of a chiral quaternary stereogenic center was reported. The reactions were conducted under two different conditions: in toluene in the presence of **C1c** and trifluoracetic acid (TFA); or in water in the presence of the perchloric acid salt of **C1a** [14]. The choice of the proper acid to use as a co-catalyst was crucial for this reaction; in organic solvents, only trifluoroacetic acid secured good yields in the reaction, while in the presence of other tested acids, the reaction was extremely slow (chloroacetic acid, *p*-nitrobenzoic acid, HCl) or only the polymerization of the diene was observed (triflic or perchloric acid). The respective adducts were formed with excellent *exo-endo* and enantioselectivities (Scheme 5).

4,4,4-Trifluorocrotone aldehyde was used as a dienophile in the presence of 10 mol% of **C1a** and HClO_4_ as the co-catalyst. Its addition to cyclopentadiene had a good yield and moderate diastereoselectivity (74% and 72:28 *endo*:*exo*, respectively) and excellent enantioselectivity (99.5:0.5) for both enantiomers. Among the tested acyclic dienes, the best results were obtained for isoprene—a 58% yield of 97.5:2.5 er [15].

Jørgensen developed a new method for the synthesis of *trans*-Diels–Alder adducts based on the halogen effect and the newly discovered “pseudo-halogen” effect [16].

**Scheme 6:**
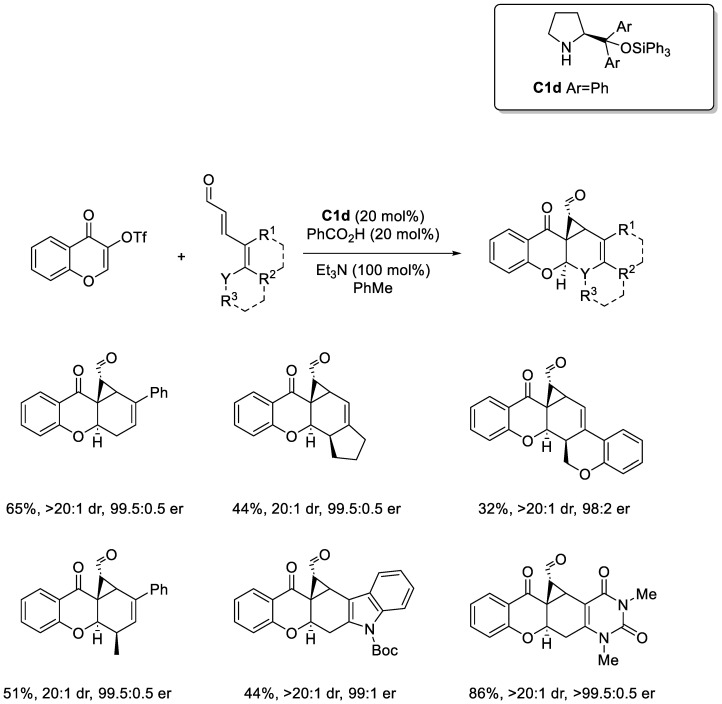
Synthesis of functionalized *trans*-Diels–Alder adducts.

The halogen-enhanced *endo*-transition state was further improved by replacing the halogen atom with a triflate moiety, a “pseudo-halogen”. The subsequent S_N_2 reaction led to the inversion of the configuration, leading to a *trans*-substituted norcarane scaffold, a core element of many natural products. The reaction performed in the presence of the **C1d** catalyst proceeded with high diastereoselectivity and excellent enantioselectivity (selected examples are presented in Scheme 6).

**Figure 5:**
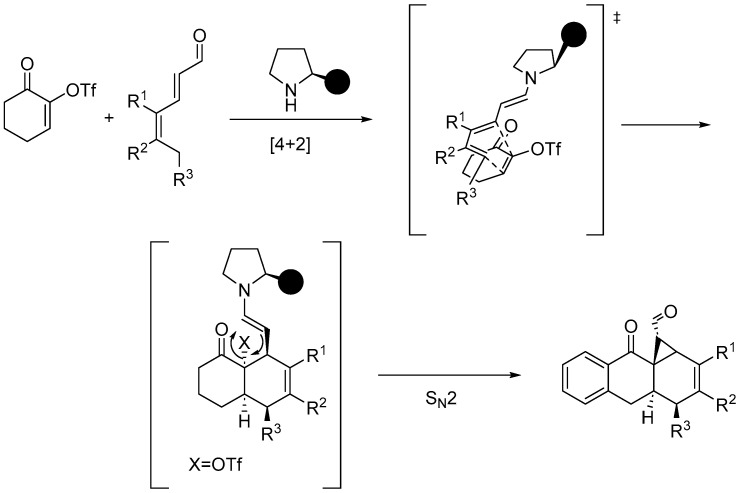
Proposed course of the reaction leading to *trans*-Diels–Alder adducts.

The proposed mechanism of the reaction was confirmed by both experimental and computational methods (Figure 5).

**Scheme 7:**
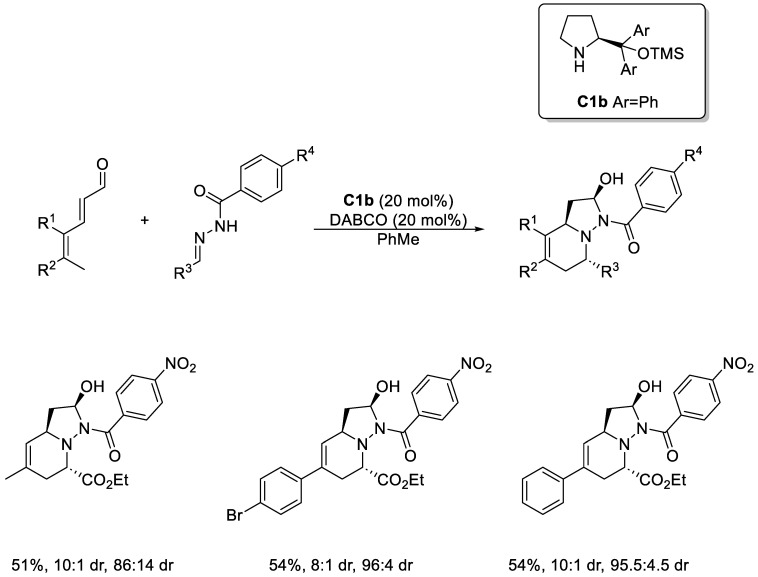
Synthesis of azaheterocycles.

The same group reported enantioselective aza-Diels–Alder reactions [17]. The cascade Diels–Alder/ring-closing reactions between acylhydrazones and in situ formed trienamines in the presence of **C1b** furnished bicyclic azaheterocycles in good yields and with high diastereo- and enantioselectivity (Scheme 7).

**Figure 6:**
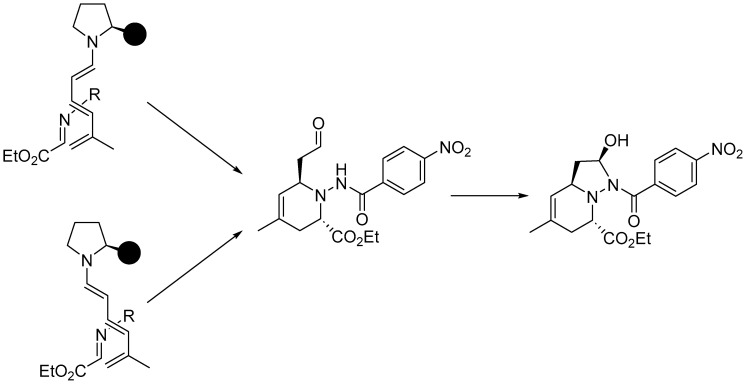
Mechanism of cascade Diels–Alder/ring-closing reactions.

The proposed mechanism of this process is presented in Figure 6.

The in situ formed trienamines were reacted with ethyl cyanophenyl acrylate as dienophile to yield tetrahydrocarbazoles. The highest selectivity, up to 92:8 dr and 99:1 er, was observed for the **C1d** catalyst [18].

**Scheme 8:**
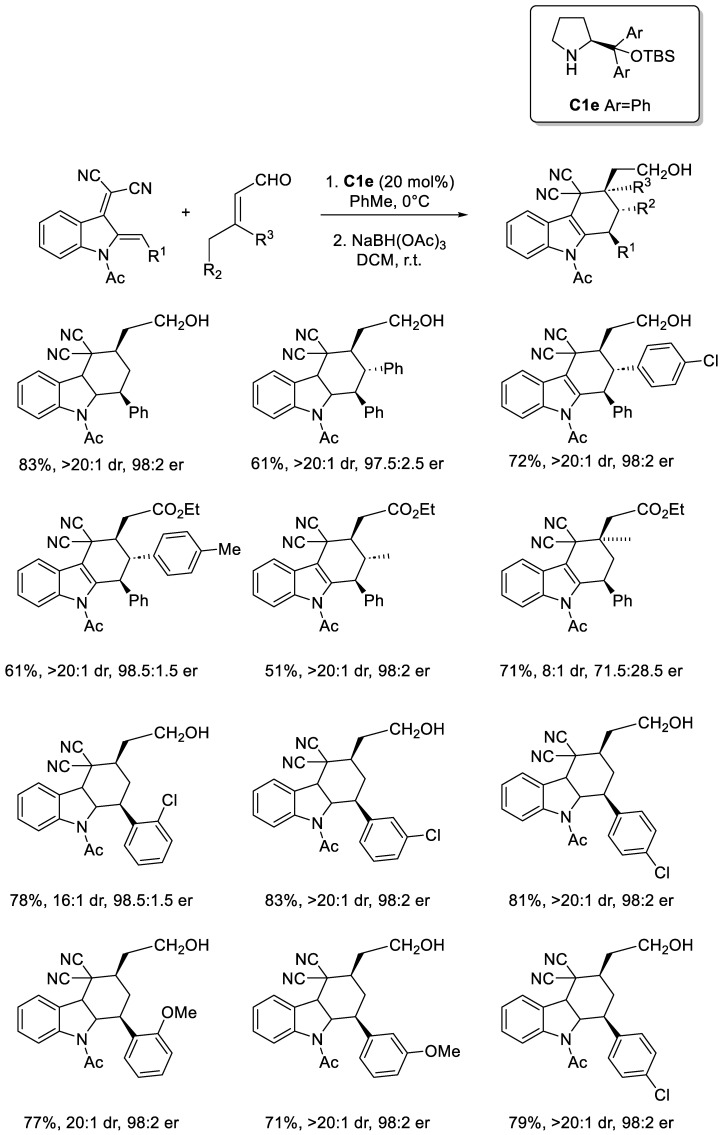
Synthesis of tetrahydocarbazoles by DA reaction of 2,3-indole dienes with α,β-unsaturated aldehydes.

Another approach to the synthesis of tetahydrocarbazoles was described by Gu et al. [19]. The novel indole dienes were subjected to [4+2] cycloaddition with α,β-unsaturated aldehydes to yield tetrahydrocarbazoles. The best enantiodiscrimination of up to 99:1 er was observed in the presence of the **C1e** catalyst (Scheme 8).

**Figure 7:**
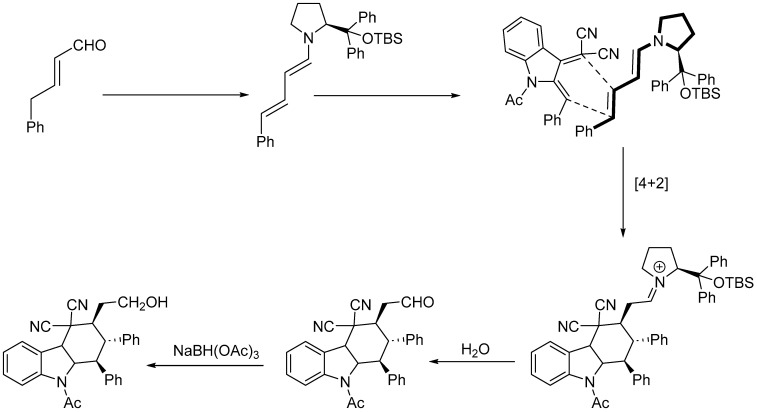
Mechanism for the formation of the tetrahydrocarbazoles.

The plausible mechanism of this transformation is presented in Figure 7.

**Scheme 9:**
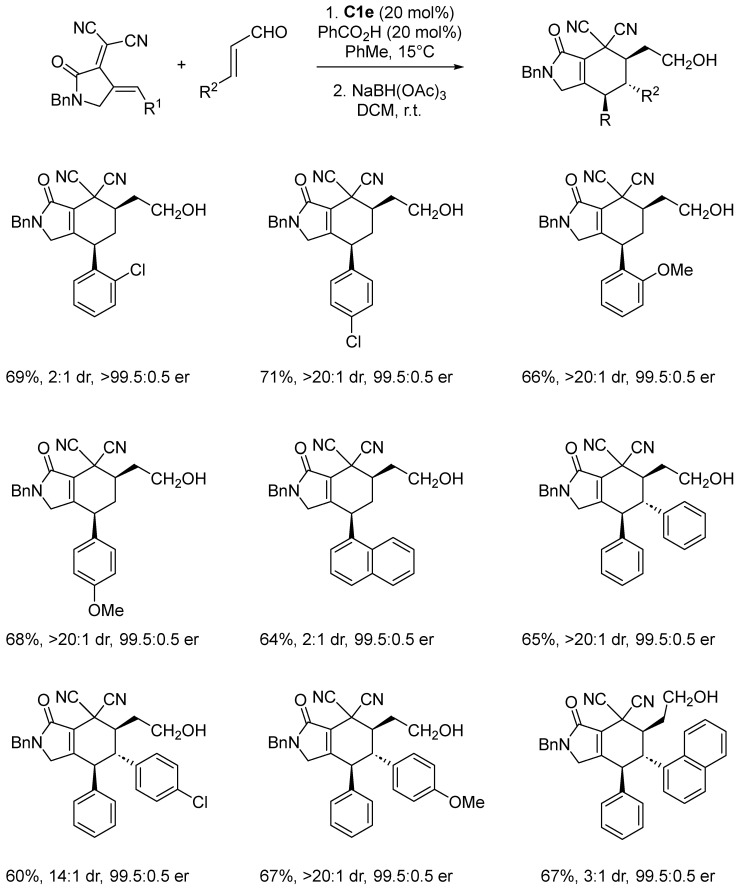
DA reaction of pyrrolidine dienes with α,β-unsaturated aldehydes in the presence of **C1e**.

The same approach was used for the highly enantioselective reaction of pyrrolidine dienes with α,β-unsaturated aldehydes in the presence of **C1e** (20 mol%) [20]. The reaction proceeds in a manner analogical to that presented in Figure 7. Selected results are presented in Scheme 9.

**Scheme 10:**
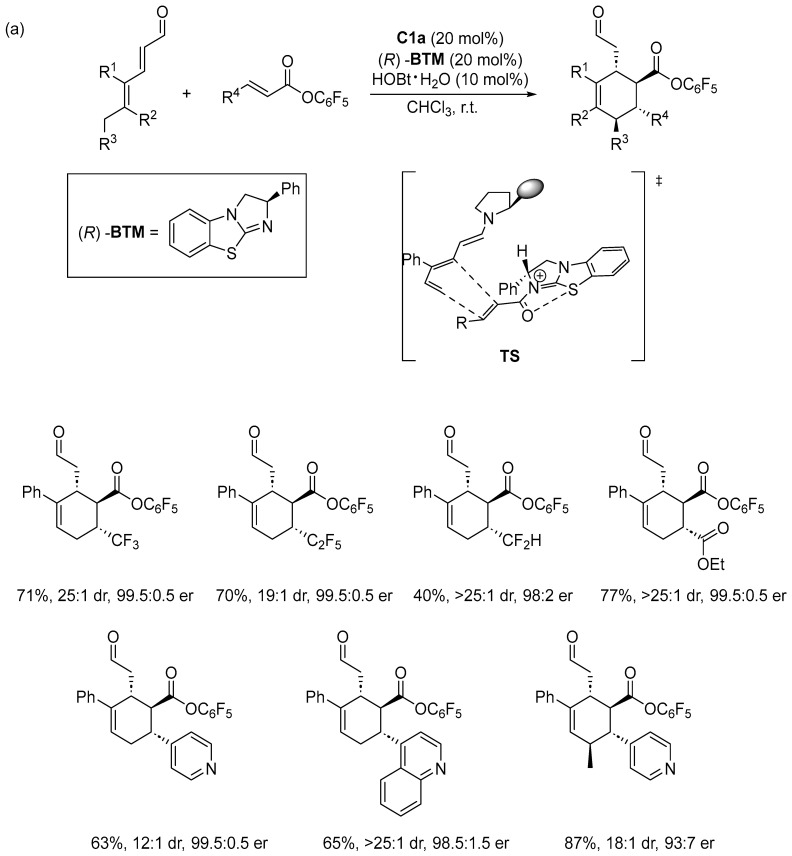

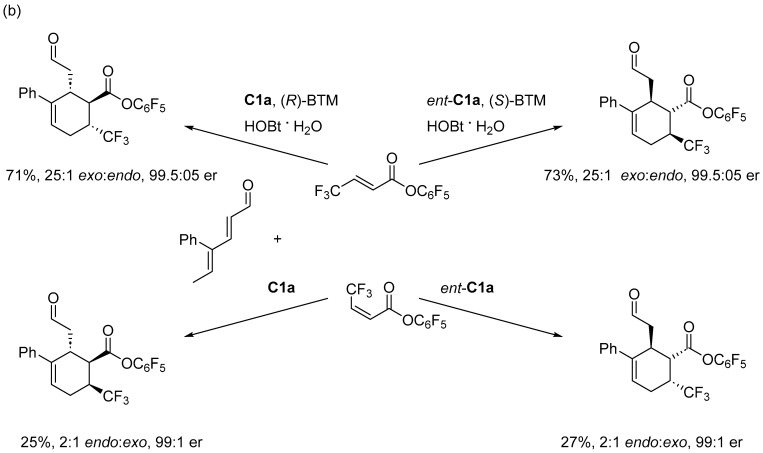
Highly selective Diels–Alder reactions between 2,4-dienals and α,β-unsaturated esters promoted by the chiral thiourea in the presence of 20 mol% of **C1a** (**a**) and the influence of the configuration of the double bond (**b**).

Highly selective Diels–Alder reactions between 2,4-dienals and α,β-unsaturated esters were promoted by the chiral thiourea BTM in the presence of 20 mol% of **C1a** [21]. This approach allows for the simultaneous activation of dienal as a chiral trienamine and an α,β-unsaturated ester as a chiral α,β-unsaturated acylammonium derivative. The activated dienophile is formed by the reaction of benzotetramisole (BTM) with a pentafluorophenyl butanoate, in which BTM readily displaces the perfluorophenoxide moiety of the ester substrate to create a chiral α,β-unsaturated acylammonium intermediate (see TS in Scheme 10). Both enantiomers of BTM were tested, and the results clearly indicate that the choice of the (*R*)- or (*S*)-BTM is important, as the (*R*)- BTM forms a matched pair with **C1a** and enhanced diastereoselectivity is observed. No such effect was visible in the presence of (*S*)-BTM. The addition of 1-hydroxybenzotriazole hydrate, which was earlier reported to increase the catalytic turnover for Lewis base catalysts, further improved the diastereoisomeric ratio of adducts. The use of enantiomeric catalytic pair *ent*-**C1a** and (*S*)-BTM lead to enantiomeric adducts with no loss of selectivity. Additionally, performing the reaction of (*Z*)-dienophile in the absence of BTM allows for the synthesis of C3-C4 *cis*-substituted adducts, albeit with a diminished chemical yield and diastereoselectivity (Scheme 10b).

The Diels–Alder reaction catalyzed by pyrrolidine catalysts (**C1**) was used as a key step in several syntheses of natural products.

**Scheme 11:**
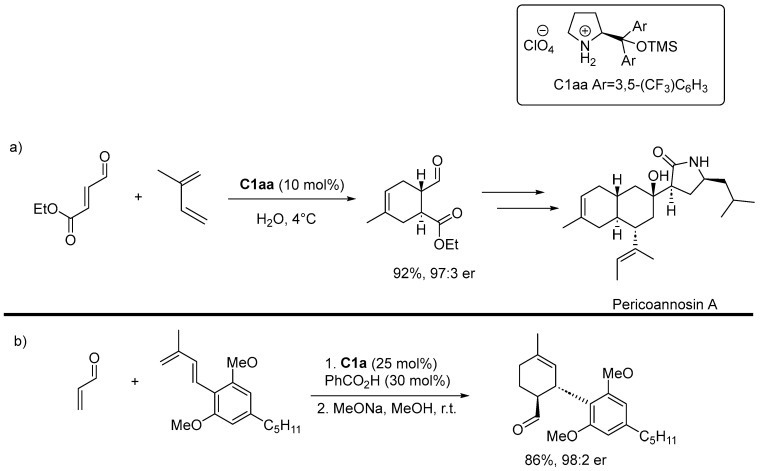
[4+2] Cycloaddition steps in the synthesis of Pericoannosin A (**a**); cannabidiols and tetrahydrocannabinols (**b**).

The reaction of ethyl (*E*)-4-oxobut-2-enoate with isoprene in the presence of 10 mol% of **C1aa** proceeded with a very good yield and enantioselectivity. The adduct was used for the formal synthesis of Pericoannosin A (Scheme 11a) [22]. Acrolein additions to specially designed diene were key steps for the enantioselective syntheses of both (−)-*trans* and *cis*-cannabidiols and Δ^9^-tetrahydrocannabinols. The best results were obtained for **C1a** and *ent*-**C1a** [23]. An example is presented in Scheme 11b.

**Scheme 12:**
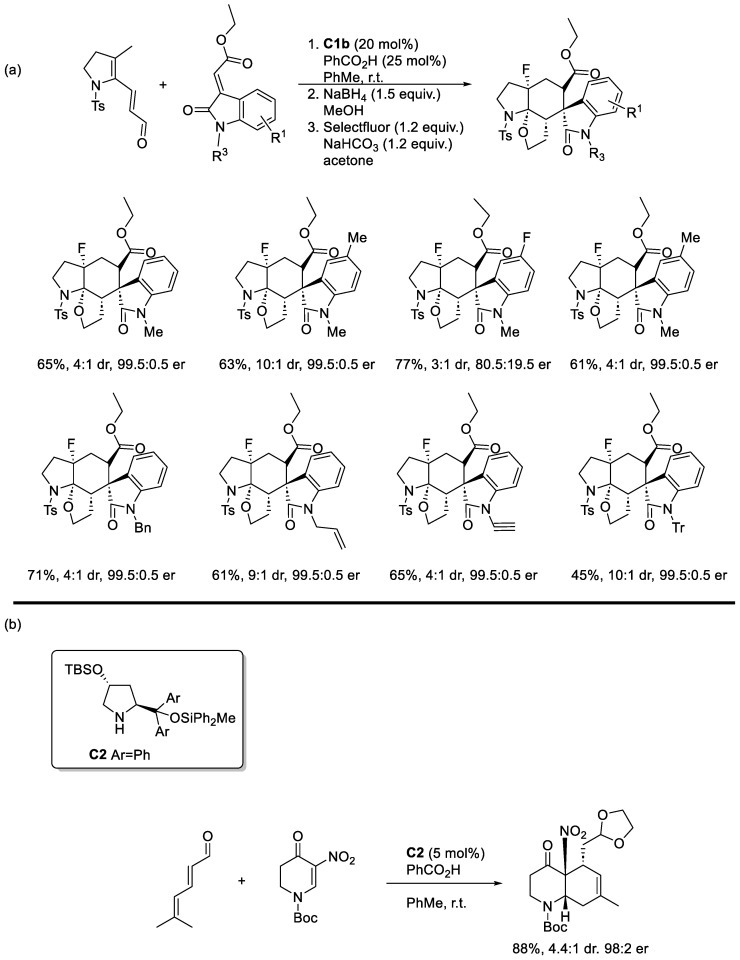
Diels–Alder reaction of prochiral dienes with α,β-unsaturated aldehyde; synthesis of tricyclic fluorooctahydrofuranoindole spirooxindoles (**a**) and synthesis of AB-ring fragment of Senepodine F (**b**).

Prasad et al. reported the enantioselective synthesis of tricyclic fluorooctahydrofuranoindole spirooxindoles starting from a Diels–Alder reaction of prochiral dienes with α,β-unsaturated aldehyde. The reaction catalyzed by Hayashi’s catalyst **C1b** (20 mol%) proceeded with good yields and diastereoselectivities, as well as excellent enantioselectivities [24]. The highly stereoselective [4+2] cycloaddition was followed by sequential reduction and fluoroetherification. Selected examples are presented in Scheme 12a.

The AB-ring fragment of Senepodine F was synthesized starting from the reaction of a 5-nitro-2,3-dihydropyridone derivative and α,β,γ,δ-unsaturated aldehyde [25]. The reaction was catalyzed by 4-hydroxyproline-derived catalyst **C2** (5 mol%) and proceeded with a very good chemical yield and excellent enantiodiscrimination (Scheme 12b).

The adduct of 1-benzyloxy-1,3-butadiene and acrolein was obtained in the presence of a MacMillan catalyst in a 56% chemical yield and with 96.5:3.5 er and was converted into perbenzylated α-carbagalactose, a fragment of the synthetic glycolipid RCAI-56 [26].

### 2.2. Amine Catalyst Other than Hayashi’s and Jørgensen’s Catalysts

**Scheme 13:**
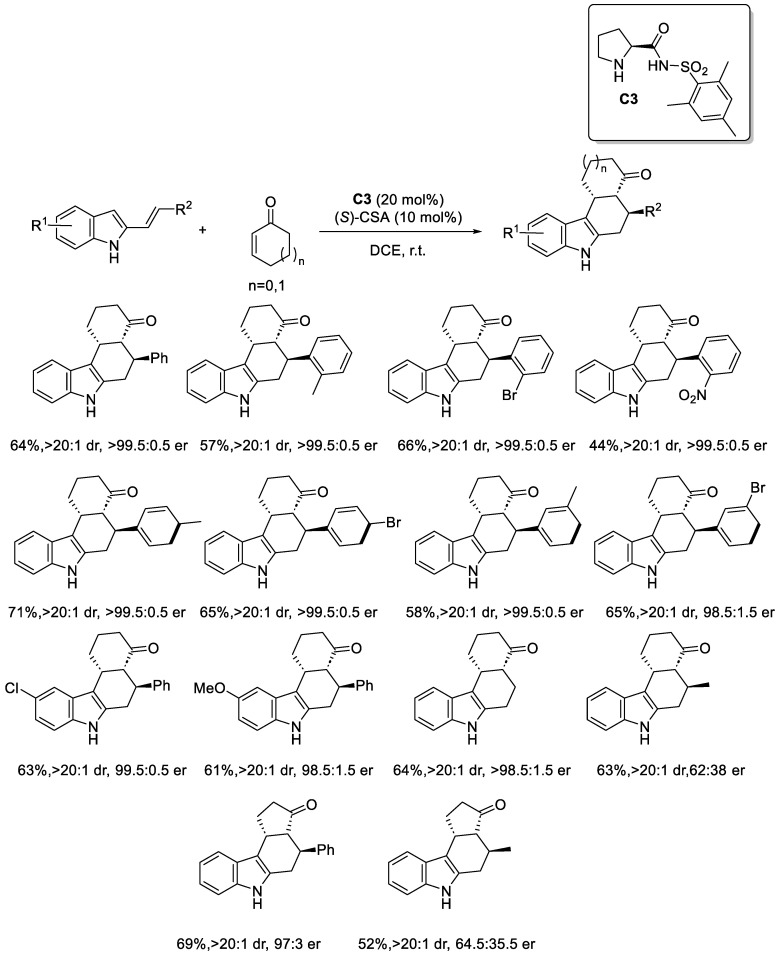
Diels–Alder reaction catalyzed by catalyst **C3**.

Proline 2,4,6-trimethylphenylsulfonamide **C3** was reported as the excellent catalyst for Diels–Alder reactions [27]. In the absence of any acidic additives, the Friedel–Crafts alkylation was the major reaction. The presence of (*S*)-camphorsulfonic acid (CSA) was crucial to achieve a very high enantioselectivity, up to 99.5:0.5 er. Selected examples are presented in Scheme 13.

**Figure 8:**
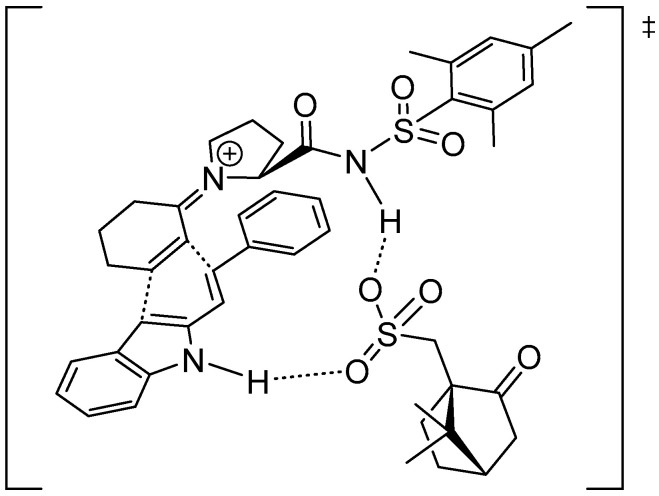
Model of transition state for the reaction of indole derivatives and cyclohexanone in the presence of **C3** and (*S*)-camphoric acid.

The authors suggest that in the absence of acid, the direct hydrogen bonding between the iminium ion and indole twist the orientation of the dienophile, making the Friedel–Crafts reaction more accessible than [4+2] cycloaddition. Upon the addition of an acid, especially a camphorsulfonic one, the new set of hydrogen bonds is formed, facilitating the Diels–Alder reaction, as presented in the suggested transition state shown in Figure 8.

**Scheme 14:**
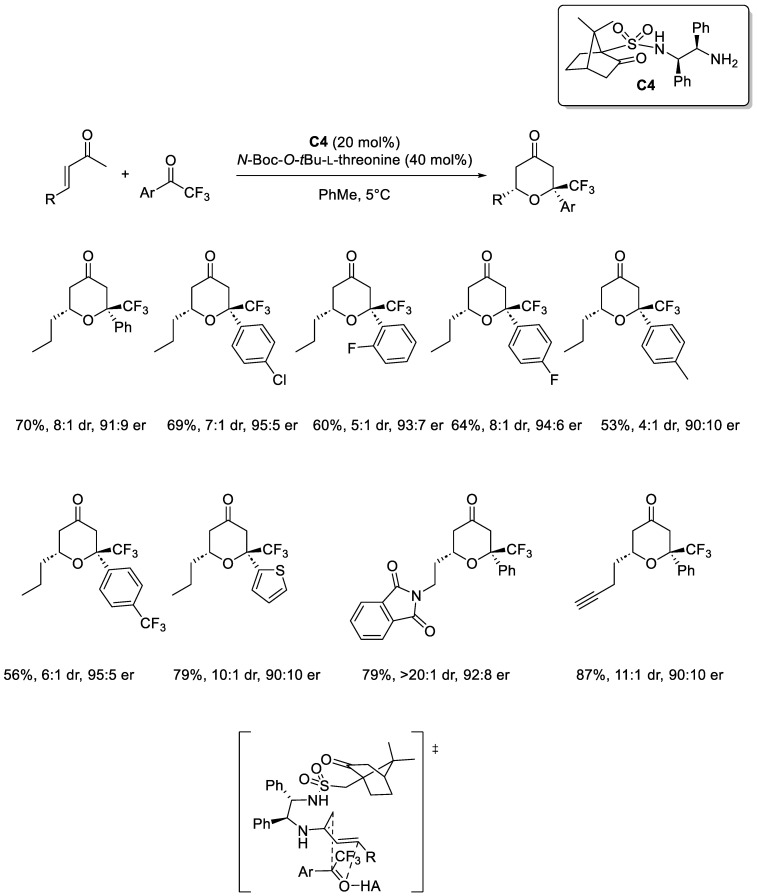
Synthesis of trifluoromethyl-substituted tetrahydropyrans in the presence of catalyst **C4**.

The sulfonamide **C4**, obtained from camphorsulfonic acid and (1*R*,2*R*)-1,2-diphenylethane-1,2-diamine, was successfully used for the [4+2] cycloaddition of enones to 2,2,2-trifluoroacetophenones [28]. The best results were obtained in the presence of an l-threonine derivative as a co-catalyst. The direction of asymmetric induction can be easily selected because both enantiomers of **C4** are available; the configuration of the threonine co-catalyst has no influence on the enantioselectivity observed. Selected examples and the proposed transition state are presented in Scheme 14.

**Scheme 15:**
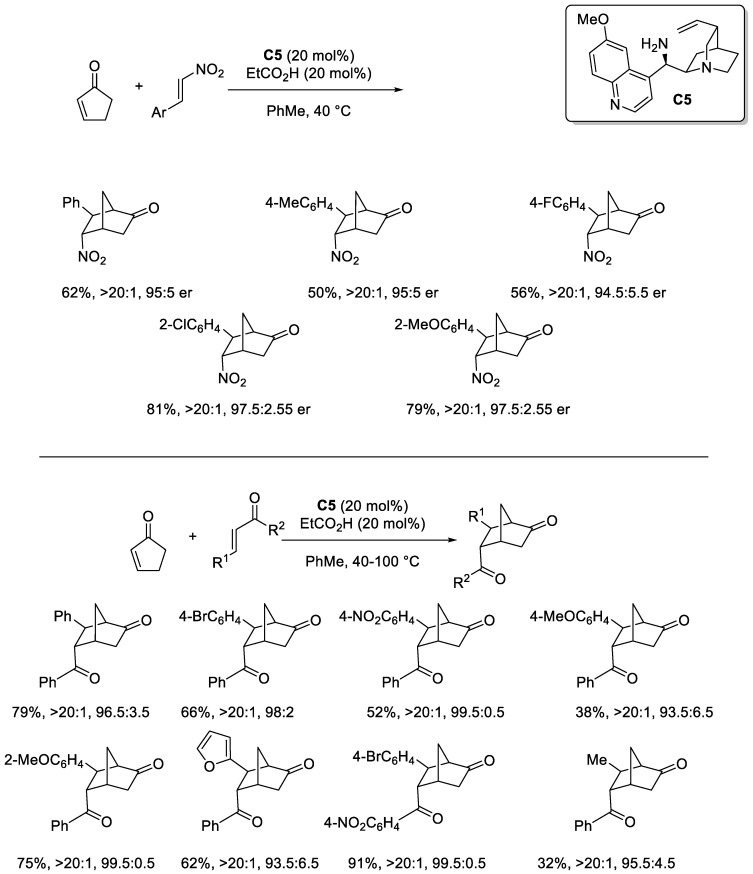
Diels–Alder reactions of nitrostyrens and chalcones catalyzed by **C5**.

Jørgensen and co-workers described highly stereoselective Diels–Alder reactions of cyclopentenone with nitrostyrens and chalcones catalyzed by quinine-derived organocatalyst **C5** (Scheme 15) [29]. The interaction of the cyclopentenone carbonyl group with the catalyst’s primary amine leads to the formation of an enamine, which yields a substituted cyclopentadiene ring reacting with dienophilic nitrostyrens and chalcones. Among several primary amines tested, **C5** had the best results. Acidic additives had only a minor influence on the stereoselectivity.

**Scheme 16:**
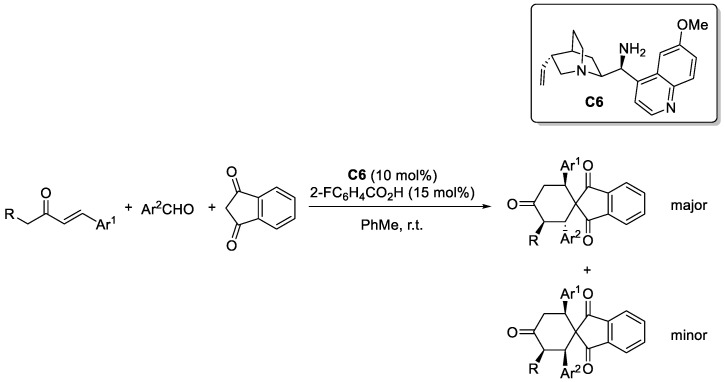
Synthesis of spiro compounds catalyzed by **C6**.

The 9-amino-9-deoxyepiquinine **C6** was used in highly stereoselective [4+2] cycloadditions of arylideneacetones and 1,3-indandione, leading to variously substituted spiro compounds [30]. The reaction was relatively insensitive to the type of aryl substituents, and in most cases, the products were obtained in good to very good yields and with excellent enantioselectivity from 97:3 up to 99.5:0.5 er (Scheme 16).

**Scheme 17:**
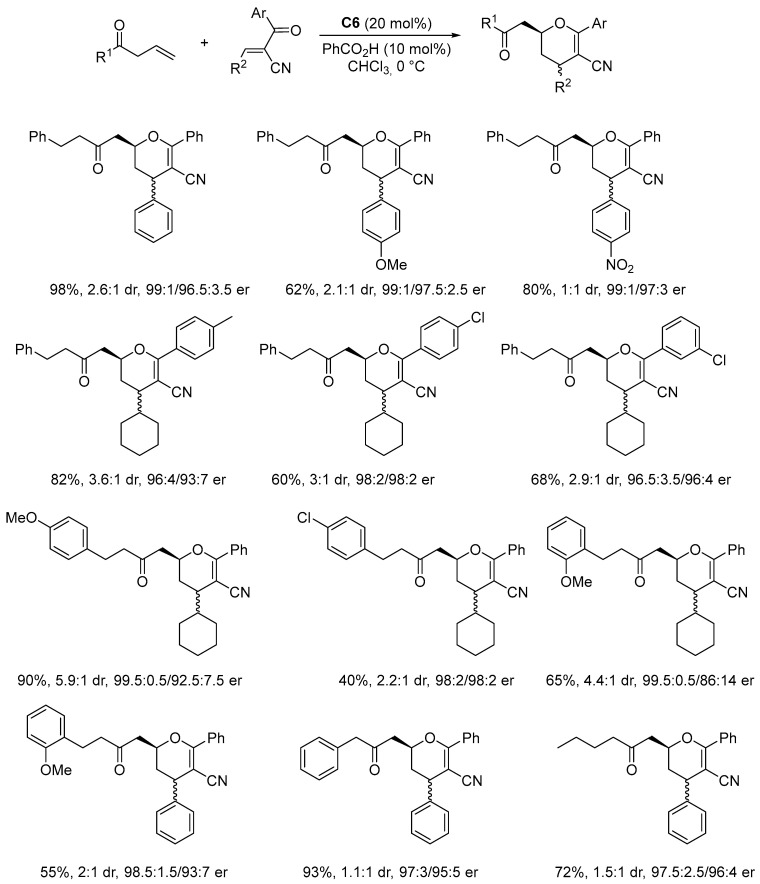
Inverse electron-demand Diels–Alder reaction catalyzed by **C6**.

The same catalyst **C6** was used for the synthesis of 3,4-dihydropyrans with stereogenic centers in positions 2 and 4 [31]. The authors reported the first organocatalytic asymmetric inverse electron-demand Diels–Alder reaction. Dienamine formation by the reaction of **C6** with deconjugated enones was the key step of this approach. While the diastereoselectivity was usually only moderate, high chemical yields and excellent enantioselectivity were observed. Selected examples are presented in Scheme 17.

### 2.3. Hydrogen Bonding in Organocatalyzed Diels–Alder Reactions

Chiral catalysts, in which the basic motive to ensure high enantiomeric excesses is the possibility of forming one or more hydrogen bonds, have become an extremely valuable tool in the hands of organic chemists in recent years.

The role of the H-bond donor was proven to be crucial in enantioselective organocatalytic synthesis. The hydrogen bonds control the facial approach during carbon–carbon bond formation and lower the barrier of activation by stabilizing charge accumulation in the transition state. Among them, chiral urea and thioureas, squaramides, and Brønsted acids are among those predominantly reported. In this section, we will discuss the latest reports on the use of catalysts from this group.

#### 2.3.1. Urea and Thiourea Catalysis

Urea and thiourea are weakly acidic and the hydrogen bonds formed are not overwhelmingly strong; however, the dual hydrogen bonding works very well for both the activation of reactants and stabilization of the transition state.

While most of the reported highly enantioselective reactions performed in the presence of (thio)urea catalysts take advantage of the chiral environment provided by Cinchona alkaloids, some recent findings show that even much simpler compounds are very efficient.

**Scheme 18:**
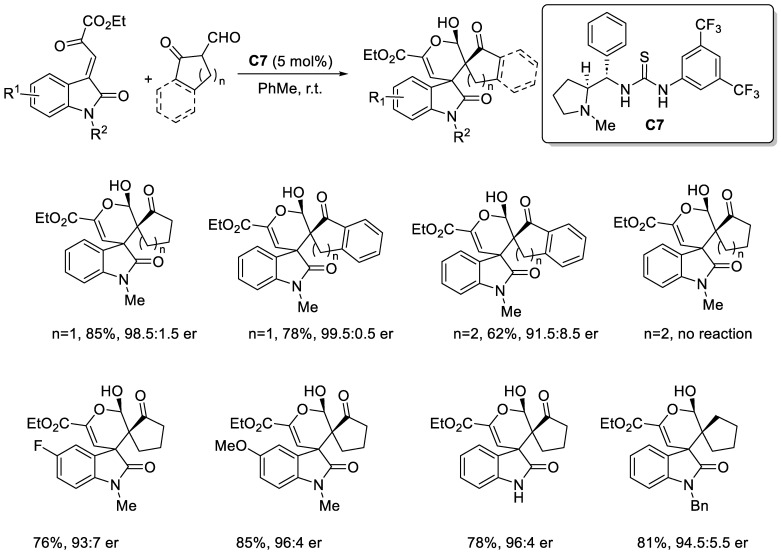
Inverse electron-demand Diels–Alder reaction catalyzed by the chiral thiourea **C7**.

The proline-derived thiourea **C7**, bearing a popular and very efficient 3,5-ditrifluoromethylphenyl moiety, was successfully used as a catalyst in an inverse electron-demand Diels–Alder reaction, yielding products with two adjacent spiro rings with a very good enantioselectivity [32]. Selected examples are presented in Scheme 18.

**Scheme 19:**
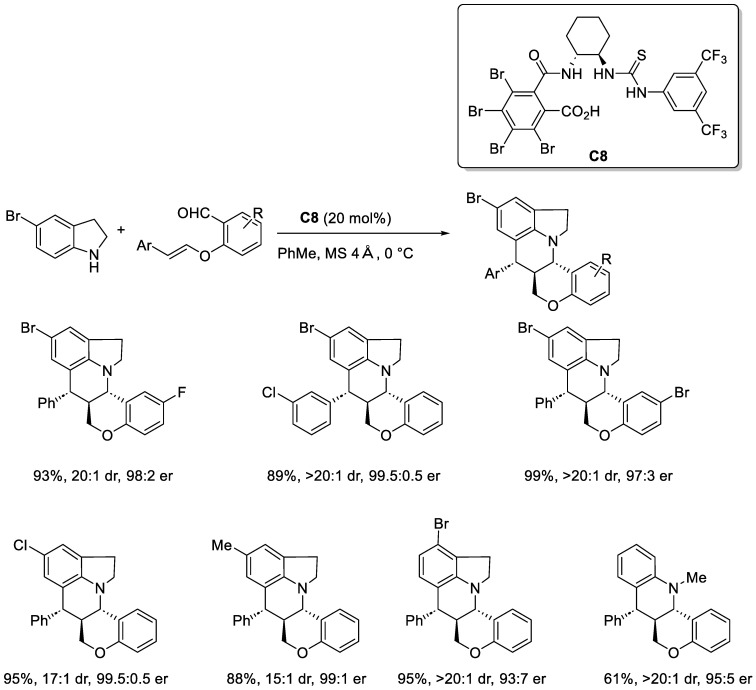
Intramolecular aza-Diels–Alder reaction.

The combination of a chiral Brønsted acid, with its chirality coming from *trans*-diamine cyclohexane (DACH), and the thiourea moiety was very effective in an intramolecular aza-Diels–Alder reaction [33]. Pentacyclic adducts (selected examples are presented in Scheme 19) were obtained with high to excellent enantioselectivities.

**Scheme 20:**
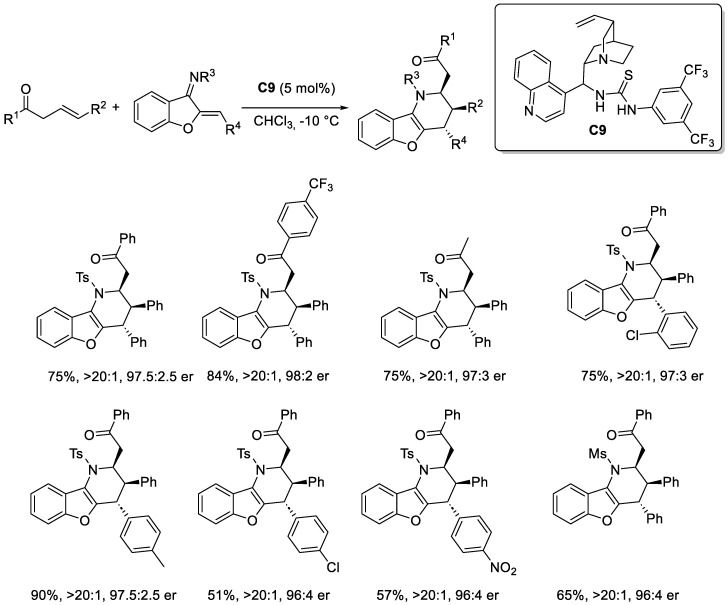
**C9** catalyzed inverse electron-demand hetero-Diels–Alder reaction of deconjugated ketones.

Thiourea catalyst **C9** derived from cinchonidine catalyzed another inverse electron-demand hetero-Diels–Alder reaction of deconjugated ketones [34]. The reaction provided polycyclic benzofuran derivatives with a tetrahydropyridine moiety, as shown in Scheme 20.

**Figure 9:**
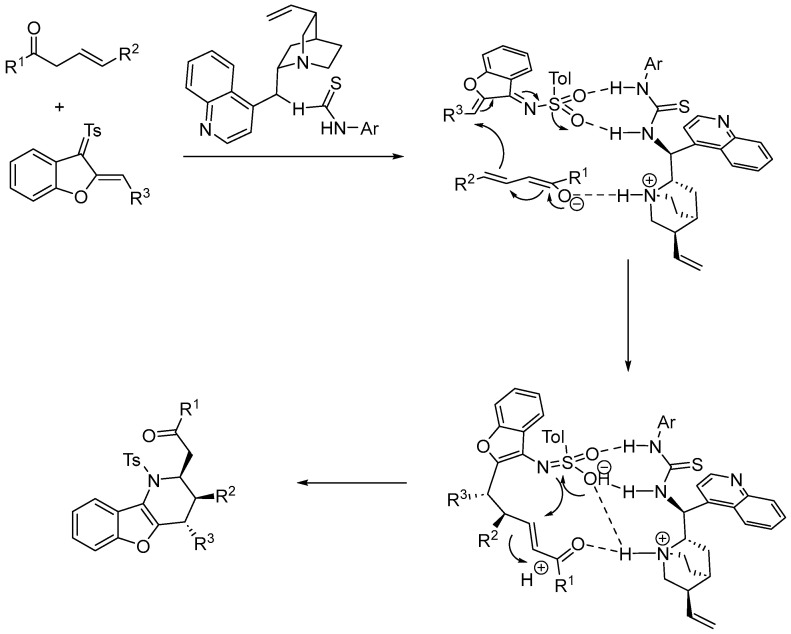
The mechanism of another inverse electron-demand hetero-Diels–Alder reaction of deconjugated ketones.

According to authors’ suggestions for reaction mechanisms, the formation of the aromatic heterocyclic ring is the driving force of the reaction, as depicted in Figure 9.

**Scheme 21:**
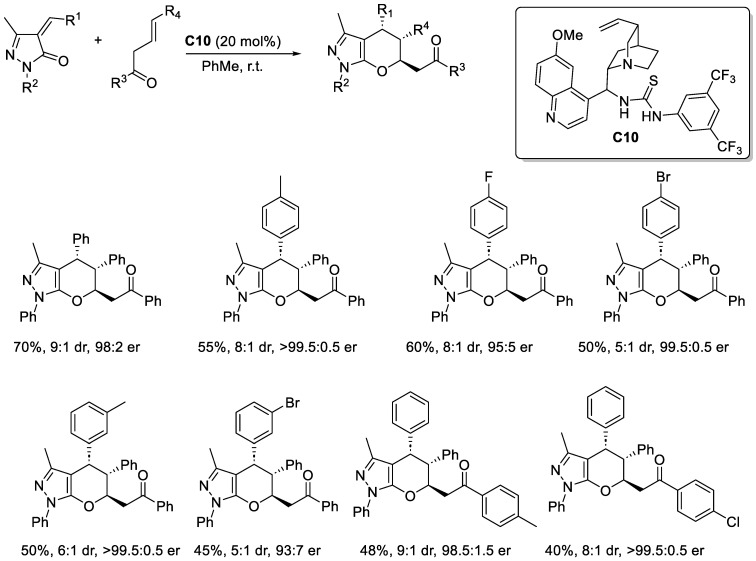
Inverse electron-demand Diels−Alder reaction of unsaturated pyrazolones with deconjugated ketones catalyzed by **C10**.

**Figure 10:**
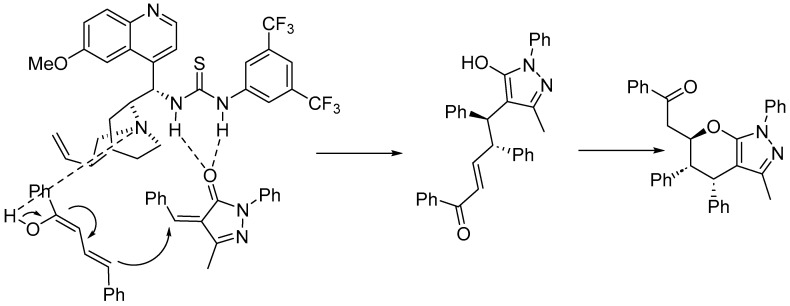
The plausible mechanism inverse electron-demand Diels−Alder reaction of unsaturated pyrazolones with deconjugated ketones.

The first inverse electron-demand Diels−Alder reaction of unsaturated pyrazolones with deconjugated ketones was performed in the presence of quinine-derived thiourea **C10** [35]. The reaction is relatively insensitive to the substitution pattern of R^1^ and R^3^ aryl groups; the best results were obtained for *ortho*- and *meta*-methyl-substituted phenyls as R^1^ and *para*-fluoro- and *meta*-chloro-substituted phenyls as R^3^ (Scheme 21). The mechanism of the reaction, as proposed by authors, is presented in Figure 10.

**Scheme 22:**
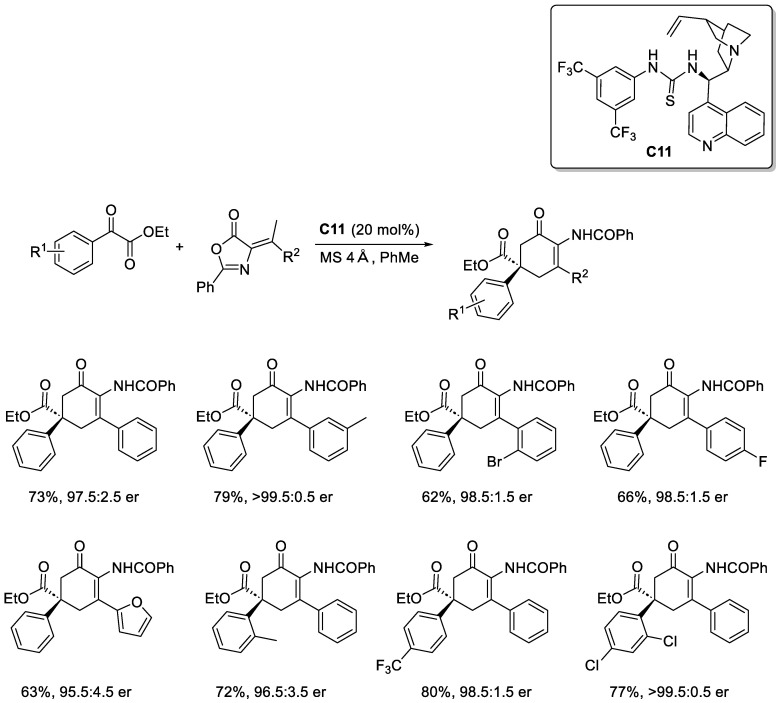
Hetero-Diels–Alder reaction of olefinic azlactones and α-ketoesters catalyzed by **C11**.

Cinchonine-derived thiourea catalyst **C11** efficiently promoted the hetero-Diels–Alder reaction of olefinic azlactones and α-ketoesters (Scheme 22) with very good to excellent enantioselectivities. The reactions of α,β-unsaturated esters were slightly less selective [36]. The selectivity of the reaction was increased when bulky substituents were used, but at the expense of the chemical yield.

**Figure 11:**
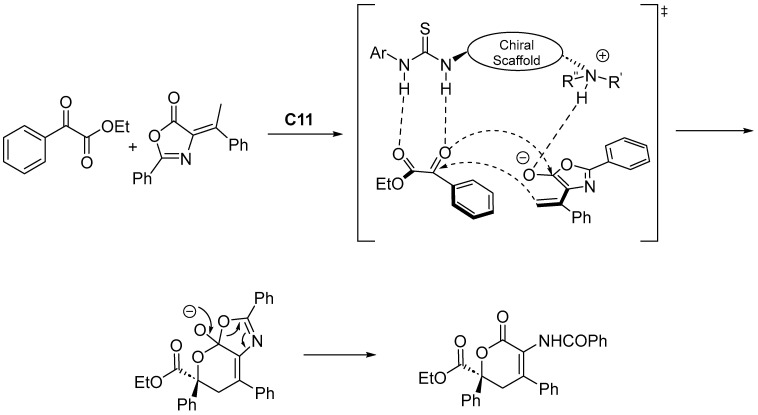
Possible reaction pathway of hetero-Diels–Alder reaction of olefinic azlactones and α-ketoesters in the presence of catalyst **C11**.

The possible transition state and the reaction pathway are presented in Figure 11.

**Scheme 23:**
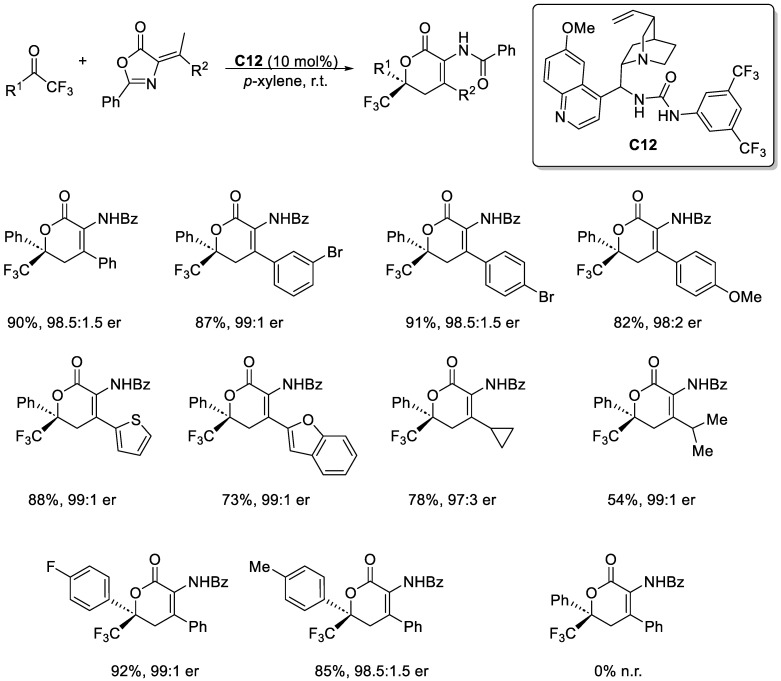
Hetero-Diels–Alder reaction of olefinic azlactones and trifluoromethyl aryl ketones catalyzed by **C12**.

Another example of [4+2] cycloaddition that takes advantage of the in situ diene’s formation was described by Singh and co-workers [37]. The addition of trifluoromethyl aryl ketones was catalyzed by quinine derivative **C12**, in this case, with the urea moiety, which provided the best conversion and enantiodiscrimination (Scheme 23). Trifluoromethyl aryl ketones with both electron-withdrawing and electron-donating substituents reacted with high selectivity, albeit the latter were less reactive. Trifluoromethyl alkyl ketones do not react under these conditions.

**Figure 12:**
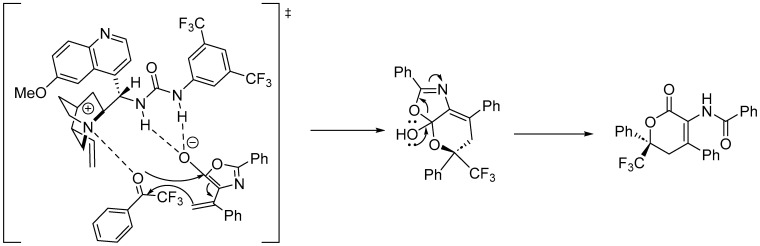
Transition state for the DA reaction catalyzed by **C12**.

The plausible transition state and reaction pathway are presented in Figure 12.

**Scheme 24:**
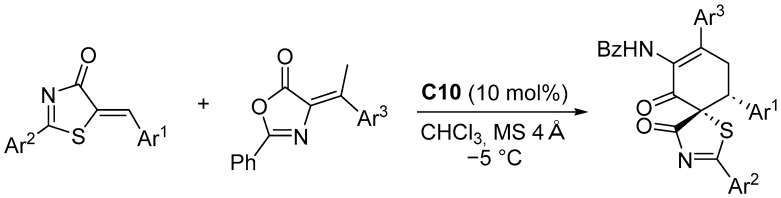
Asymmetric organocatalytic Diels−Alder reaction of olefinic azlactones with unsaturated thiazolones.

The alkylidene azlactones were used as dienes in the **C10**-catalyzed DA reaction with alkylidene thiazolone, yielding spirocyclohexenone thiazolones with good chemical yields and moderate to high enantioselectivities (Scheme 24) [38].

**Scheme 25:**
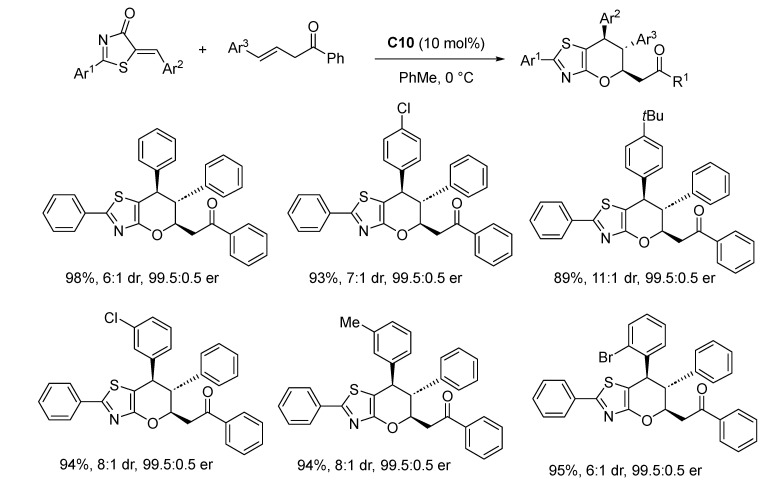

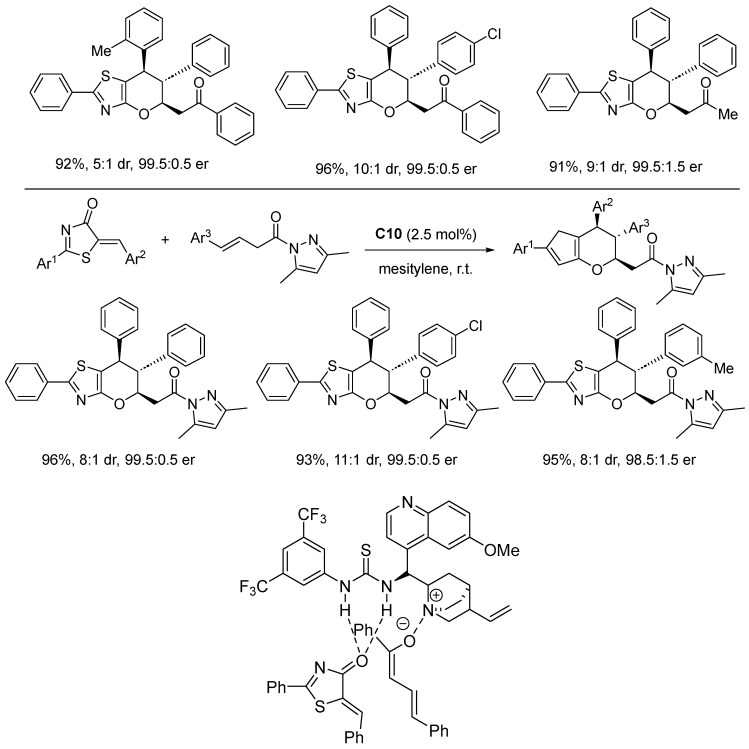
Inverse electron-demand DA reaction of 5-alkenyl thiazolones with β,γ-unsaturated carbonyl compounds in the presence of catalyst **C10**.

The quinine-derived thiourea catalyst **C10** has been successfully used in inverse electron-demand DA reactions of 5-alkenyl thiazolones with β,γ-unsaturated carbonyl compounds—ketones and amides [39]. In most cases, the stereoselectivity was excellent, with an enantiomeric ratio of 99.5:0.5. Selected examples and the proposed transition state are presented in Scheme 25.

**Scheme 26:**
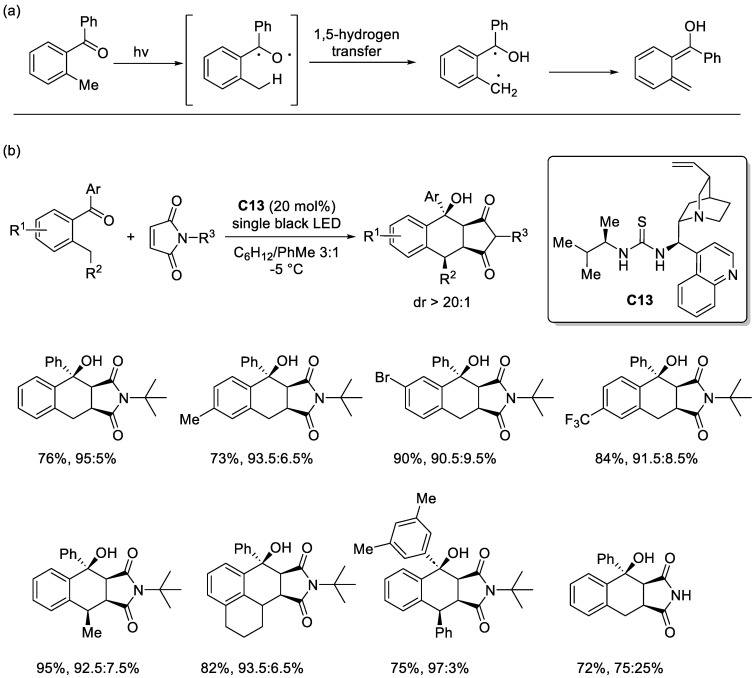
The photoenolization of acetophenones leading to hydroxy-*o*-quinodimethanes (**a**) and the photomediated enantioselective Diels–Alder reaction catalyzed by **C13** (**b**).

Enantioselective radical reactions have become one of the most valuable tools in asymmetric organic synthesis in recent years [40]. Dell’Amico et al. combined the photoenolization of acetophenones leading to hydroxy-*o*-quinodimethanes (Scheme 26a) with chiral organocatalysis by quinine-derived thiourea [41]. In the presence of **C13**, the DA reaction proceeded with good to very good chemical yields and enantioselectivities. The major obstacle in this reaction is the formation of the photoenol, which can react in the background racemic process. The authors found that the chiral catalyst had a dual role in the investigated DA reaction. The presence of the tertiary nitrogen atom in the quinuclidine moiety inhibits the formation of the photoenol, thus diminishing the background racemic reactions. On the other hand, the thiourea moiety with the cinchona chiral part acts as a chiral catalyst, forming a chiral space for the enantioselective DA reaction. These factors allow for the high enantioselectivity of the process. Selected results are presented in Scheme 26b.

#### 2.3.2. Squaramide-Based Catalysis

Cinchona alkaloid-derived squaramides were proven to be another group of very efficient organocatalysts, being complimentary to (thio)urea derivatives. While the catalytic activity for both (thio)urea and squaramides is based on the formation of hydrogen bonds with reactants, there are significant differences between (thio)urea and squaramides, which highly influence their catalytic properties.

The distance between N-H groups is larger in squaramides than in thioureas or ureas—2.7 Å vs. 2.1 Å or 2.3 Å. Moreover, the N-H groups in squaramides, and not in (thio)ureas, are tilting into each other by approximately 6°. Also, as squaramides are more acidic than (thio)ureas, they can form stronger hydrogen bonds, being more active catalysts.

**Scheme 27:**
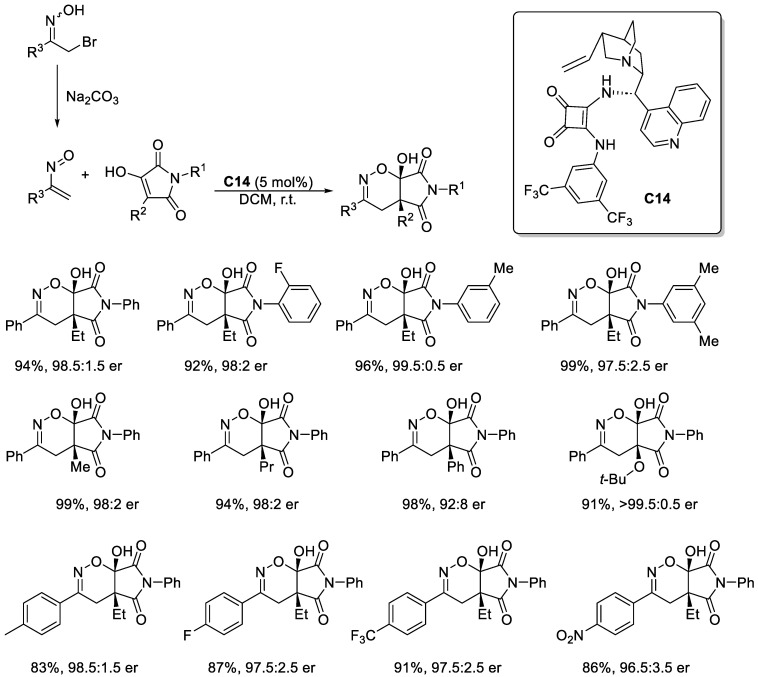
Inverse electron-demand oxa-Diels–Alder (IEDDA) in the presence of Cinchona-derived squaramide **C14**.

Catalyst **C14** proved to be significantly more effective then an analogical thiourea derivative in the HDA reaction of hydroxymalenimids with nitrosoalkenes [42]. A series of 5,6-dihydro-4*H*-1,2-oxazine succinimides was prepared with very good to excellent chemical yields and enantioselectivities (Scheme 27).

**Figure 13:**
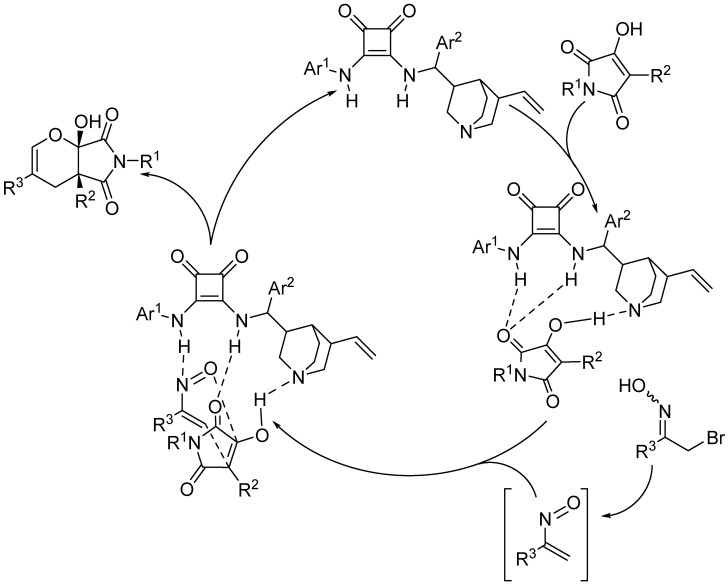
Plausible catalytic cycle for inverse electron-demand oxa-Diels–Alder in the presence of Cinchona-derived squaramide **C14**.

The proposed catalytic cycle is presented in Figure 13.

**Scheme 28:**
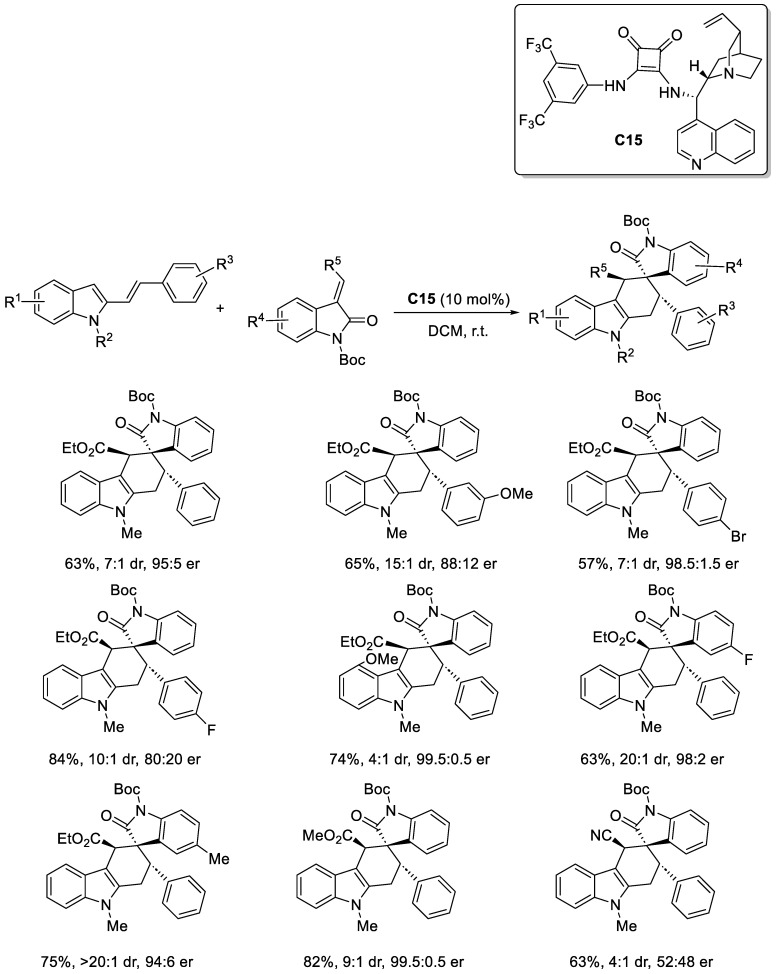
**C15**-catalyzed [4+2] cycloaddition of methyleneindolinones to 2-vinylindoles.

The cinchonidine-derived squaramide **C15** catalyzed the [4+2] cycloaddition of methyleneindolinones to 2-vinylindoles [43]. The reaction led to various carbazolespirooxindole derivatives in good yields and moderate to excellent enantioselectivities. Interestingly, the replacement of the carboethoxy group in methyleneindolinone led to a substantial improvement of the enantiomeric ratio, and the substitution with a nitrile group led to an almost racemic product (Scheme 28).

**Scheme 29:**
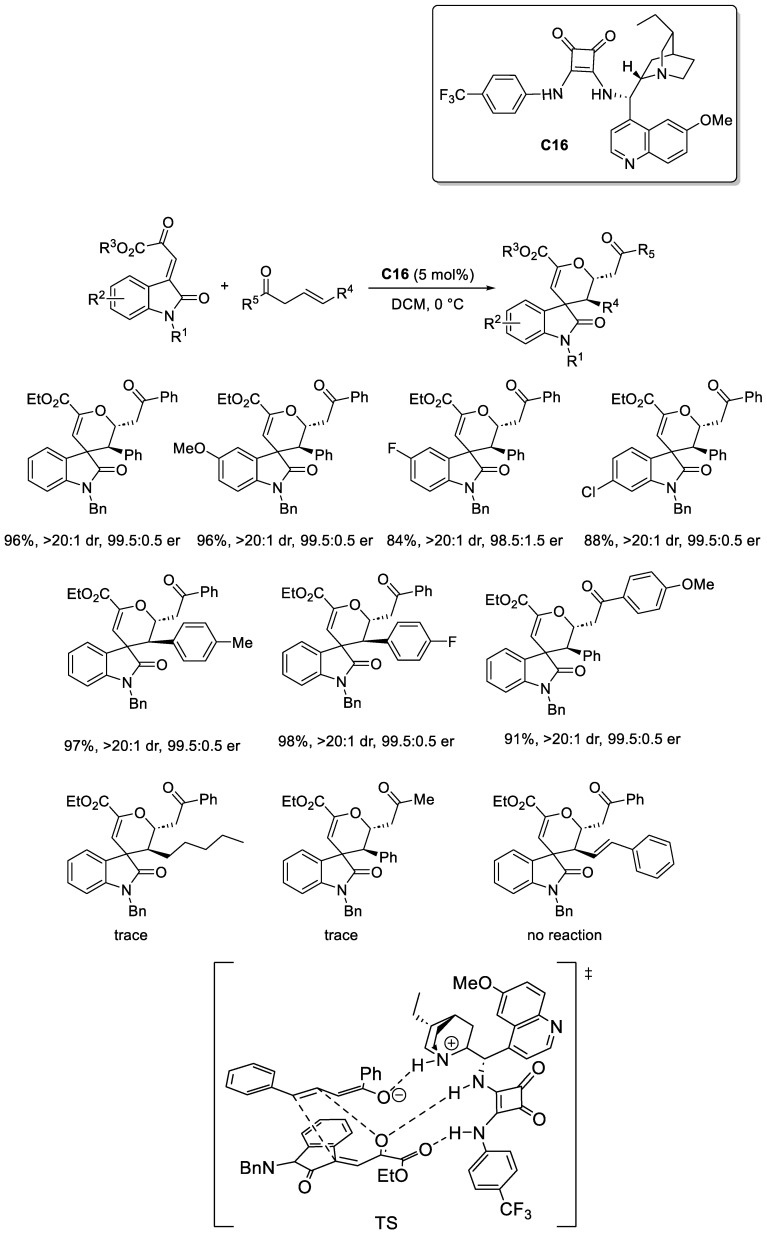
**C16**-catalyzed inverse electron-demand oxa-Diels–Alder reactions.

A series of potentially useful chiral 3,4′-pyranyl spirooxindoles was synthesized by the inverse-electron-demand oxa-Diels-Alder reactions catalyzed by Cinchona-derived squaramide **C16** [44]. The reaction proceeded with very high to excellent chemical yields and enantioselectivities. Such excellent results were achievable only for aromatic (R^4^ = R^5^ = Ar) and not for purely aliphatic deconjugated enones (Scheme 29).

#### 2.3.3. Brønsted Acid Catalysis

Acid catalysis, mainly Brønsted acid catalysis with chiral phosphoric acids (CPA), has become one of the most important tools in contemporary asymmetric synthesis. It was used in the beginning in studies on organocatalytic enantioselective DA reactions. It is still being developed, and new findings are being reported. In opposition to (thio)ureas and squaramides, they are single-hydrogen-bond donors, though they are more acidic by 20 or more pK_a_ units, which secures a strong H-bond in the transition state.

**Scheme 30:**
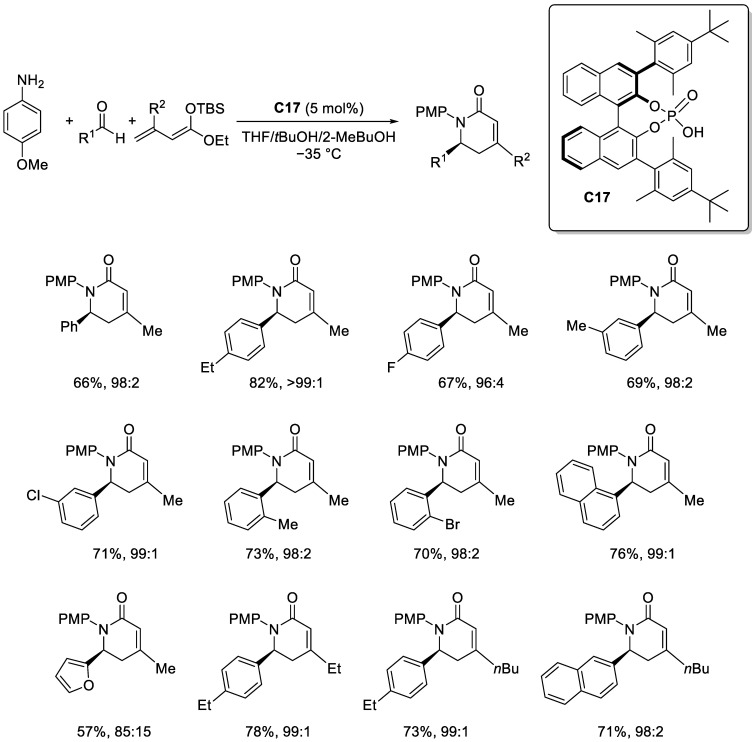
Aza-hetero-Diels–Alder reaction leading to 2-piperodinones.

Schneider and co-workers reported a three-component aza-hetero-Diels–Alder reaction of *p*-methoxyaniline, aryl aldehydes, and a vinylketene silyl acetal, performed in the presence of a very acidic catalyst, **C17** [45]. Adducts were obtained with good chemical yields and an excellent enantiomeric ratio from 96:4 up to >99:1, except for when furfural was used as the aldehyde (85:15 er). Selected examples are presented in Scheme 30.

**Scheme 31:**
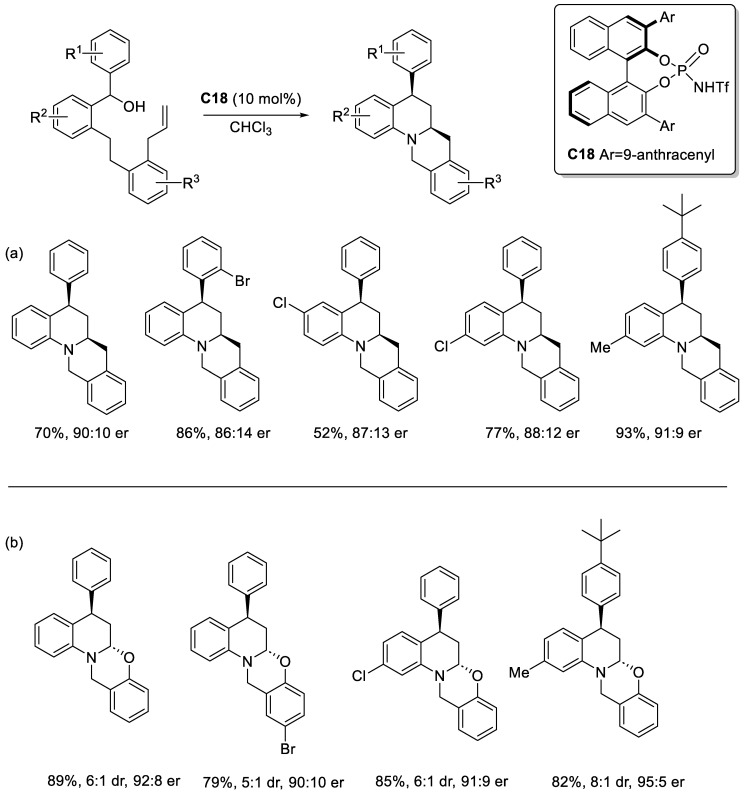
Intramolecular aza-Diels–Alder reaction of *ortho*-quinone methide imines leading to benzannulated quinolizidines (**a**) and oxazinoquinolines (**b**).

The same group reported an intramolecular aza-Diels–Alder reaction of ortho-quinone methide imines, leading to benzannulated quinolizidines and oxazinoquinolines [46]. The best results were obtained with *N*-triflyl phosphoric acid amide **C18** as the catalyst. The reactions proceeded with good to very good chemical yields and enantioselectivities. Better results were obtained with substrates bearing the enol ether moiety (Scheme 31).

**Scheme 32:**
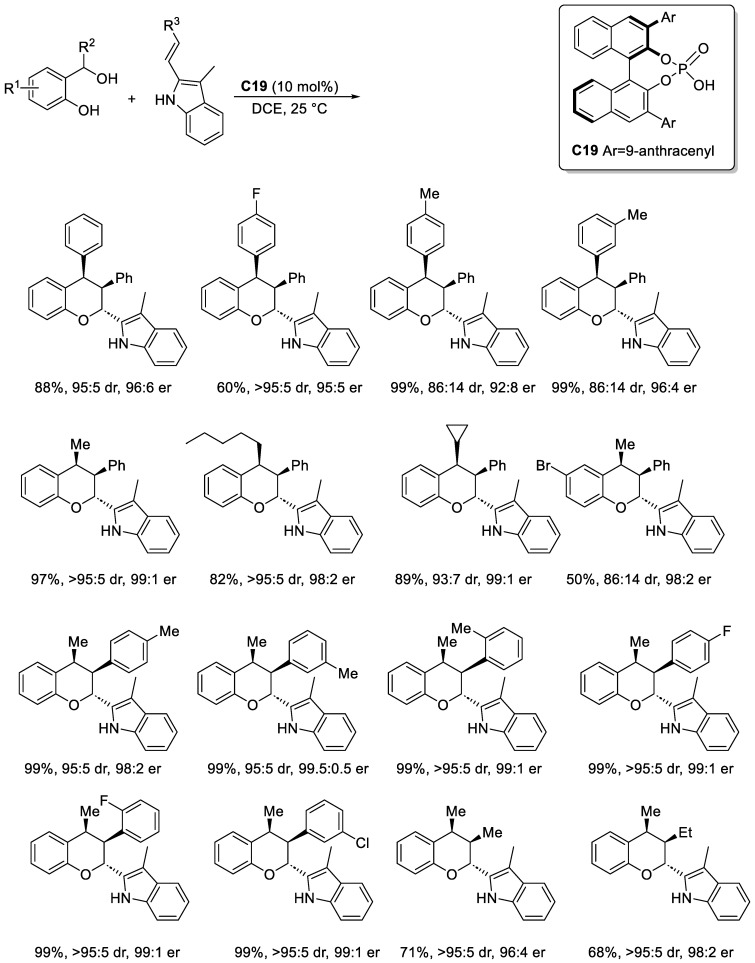
Inverse electron-demand oxa-Diels–Alder reaction catalyzed by CPA **C19**.

**Figure 14:**
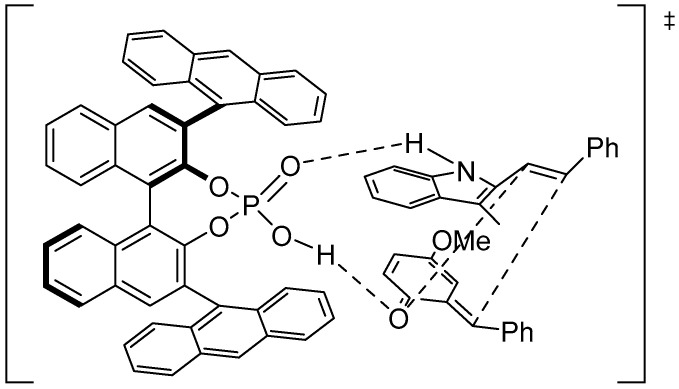
Proposed transition state for inverse electron-demand oxa-Diels–Alder reaction catalyzed by CPA **C19**.

*Ortho*-quinone methides were also used as dienes in inverse electron-demand oxa-Diels–Alder reaction with 2-vinylindoles [47]. The best results were obtained in the presence of CPA **C19**; enantioenriched chromans were obtained with very good chemical yields and stereoselectivities (Scheme 32). The DA reaction furnished adducts with excellent enantioselectivity, regardless of the type (aromatic or aliphatic groups) of the R^2^ substituent of the benzyl alcohol. The proposed transition state is presented in Figure 14.

**Scheme 33:**
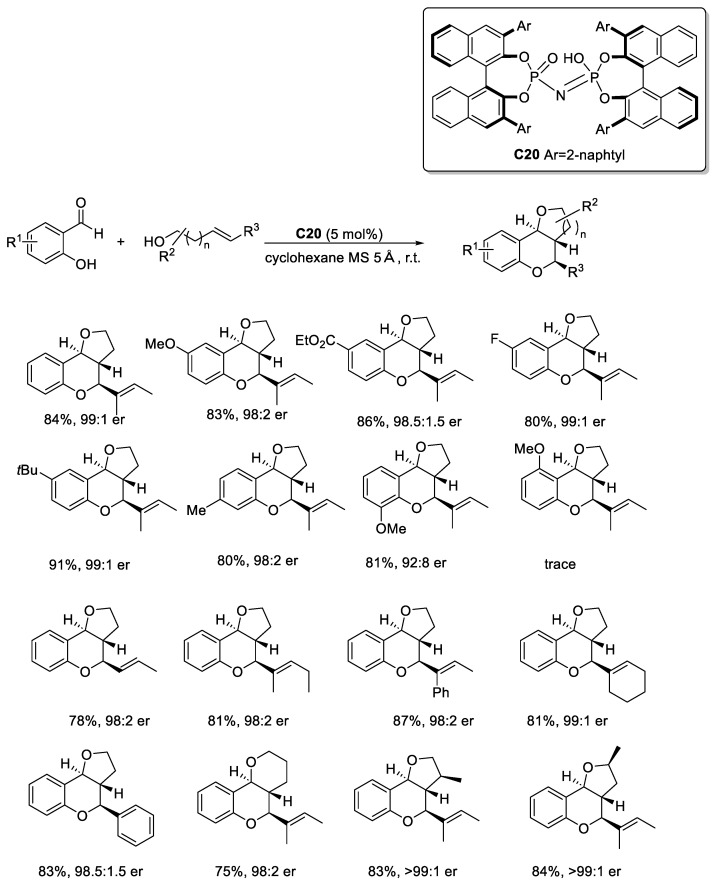
Intramolecular oxa-Diels–Alder reaction catalyzed by imidodiphosphoric acid catalyst **C20**.

An intramolecular oxa-Diels–Alder reaction catalyzed by imidodiphosphoric acid catalyst **C20** also took advantage of the easy formation of the *ortho*-quinone methide intermediate [48]. A series of furano- and chromanopyrane derivatives was obtained with good chemical yields and very high to excellent enantiomeric ratios (Scheme 33). The concerted [4+2] cycloaddition is the most probable pathway for this reaction, as confirmed by experiments.

**Scheme 34:**
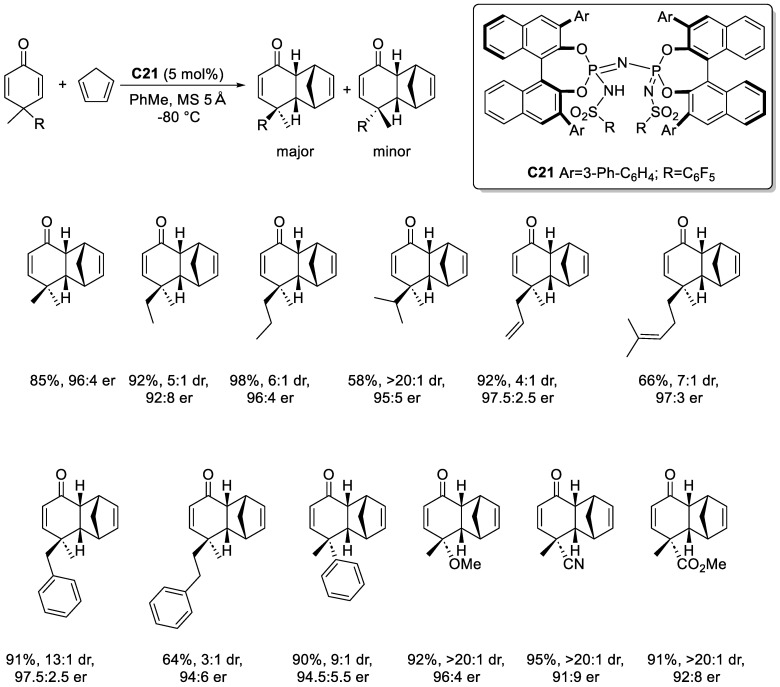
Reaction of cyclohexadienones with cyclopentadiene, catalyzed by imidodiphosphorimidate **C21**.

The same group reported the [4+2] cycloaddition of cross-conjugated cyclohexadienones with cyclopentadiene, catalyzed by imidodiphosphorimidate **C21** [49]. This elaborated chiral phosphoric acid derivative, which was highly acidic and confined, secured a very high diastereoselectivity and enantiodiscrimination (see Scheme 34).

**Figure 15:**
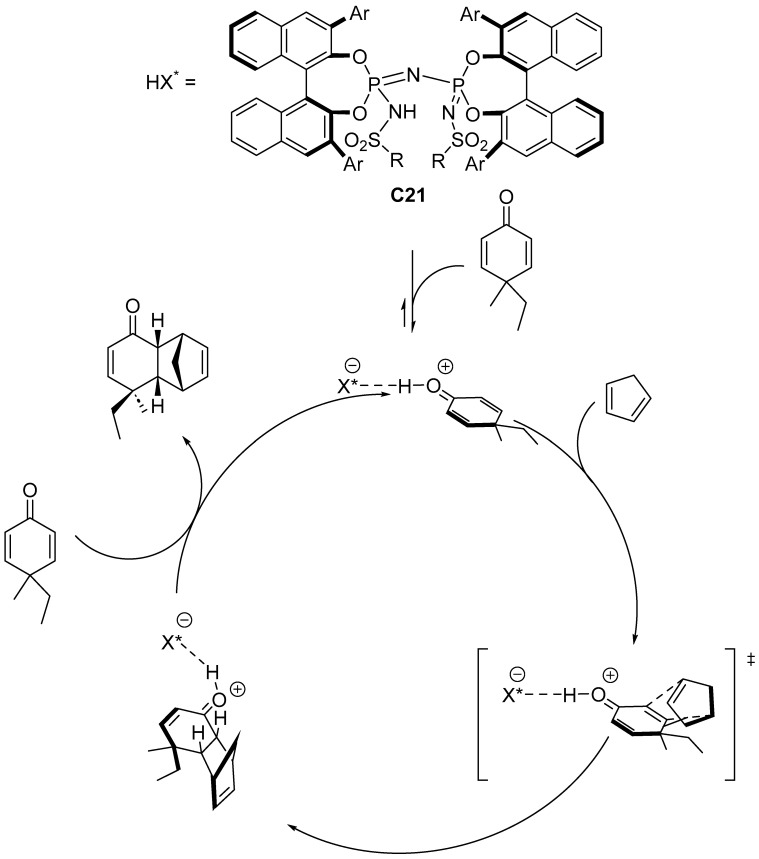
Catalytic cycle for [4+2] cycloaddition of cross-conjugated cyclohexadienones with cyclopentadiene.

Computational studies performed by authors are in very good agreement with experimental data and explain the origin of the enantio- and diastereoselectivity observed in this reaction. A plausible catalytic cycle and transition state are presented in Figure 15.

**Scheme 35:**
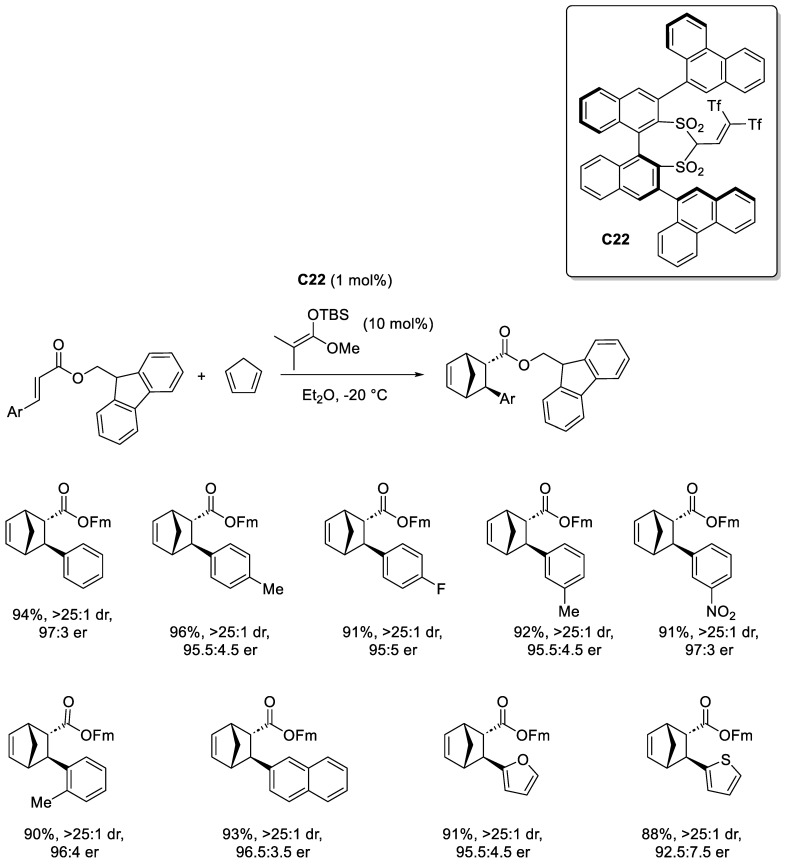
Addition of cinnamates to cyclopentadiene in the presence of silylated C-H acid.

Interestingly, the silylium ion activation of a chiral Brønsted acid was described by List and co-workers [50]. The moderately active precatalyst **C22,** upon treatment with the silylium ion source, became a very active chiral Lewis acid, efficiently catalyzing the addition of cinnamates to cyclopentadiene. Selected results are presented in Scheme 35.

**Figure 16:**
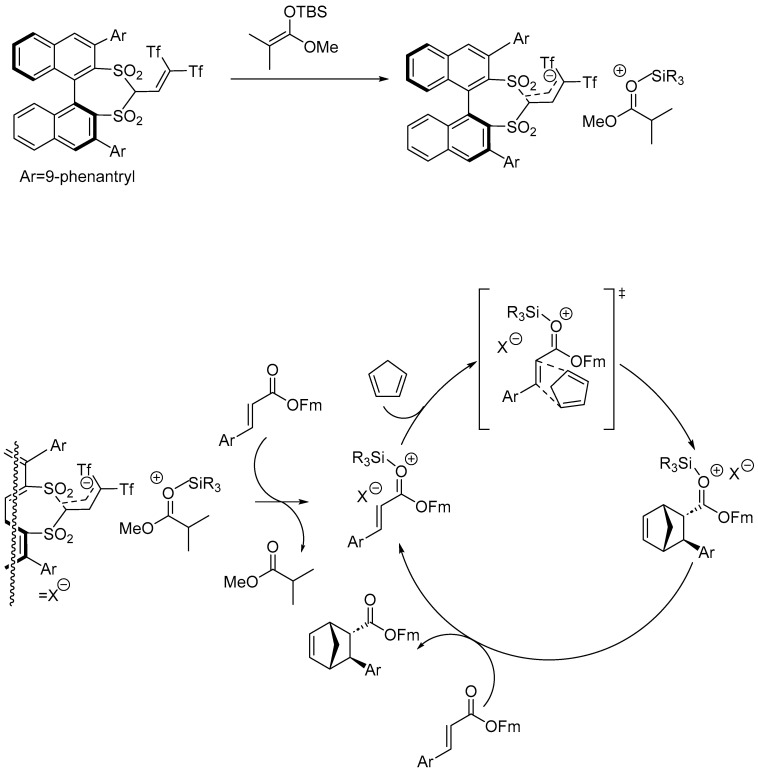
Catalytic cycle for addition of cinnamates to cyclopentadiene.

The proposed catalytic cycle is presented in Figure 16.

## 3. Conclusions

Enantioselective organocatalytic [4+2] cycloaddition has been developing rapidly in recent years, and new types of enantioselective reactions, based on novel organocatalysts, new dienophiles, and dienes compatible with organocatalysis are emerging.

Certainly, in the coming years, the scope of highly enantioselective organocatalytic [4+2] cycloadditions leading to new groups of compounds will be increasingly broad. We await the development of new methods that allow for the use of several “popular” and useful dienophiles as acrylates, crotonates, malonates, and fumarates, which should expand the scope of organocatalytic Diels–Alder reactions; such possibilities still exist, as shown by the works of Lee [21] and List [50]. There is still room for development for new, multifunctional catalysts, which can make use of at least two of the modes described above, making organocatalysis even more useful and general.
